# A Design of Experiments (DoE) Approach Accelerates the Optimization of Copper-Mediated ^18^F-Fluorination Reactions of Arylstannanes

**DOI:** 10.1038/s41598-019-47846-6

**Published:** 2019-08-06

**Authors:** Gregory D. Bowden, Bernd J. Pichler, Andreas Maurer

**Affiliations:** 1 Werner Siemens Imaging Center, Department of Preclinical Imaging and Radiopharmacy, Eberhard Karls University, Tübingen, Germany; 2 iFIT-Cluster of Excellence, Eberhard Karls University, Tuebingen, Germany

**Keywords:** Drug development, Nuclear chemistry

## Abstract

Recent advancements in ^18^F radiochemistry, such as the advent of copper-mediated radiofluorination (CMRF) chemistry, have provided unprecedented access to novel chemically diverse PET probes; however, these multicomponent reactions have come with a new set of complex optimization problems. Design of experiments (DoE) is a statistical approach to process optimization that is used across a variety of industries. It possesses a number of advantages over the traditionally employed “one variable at a time” (OVAT) approach, such as increased experimental efficiency as well as an ability to resolve factor interactions and provide detailed maps of a process’s behavior. Here we demonstrate the utility of DoE to the development and optimization of new radiochemical methodologies and novel PET tracer synthesis. Using DoE to construct experimentally efficient factor screening and optimization studies, we were able to identify critical factors and model their behavior with more than two-fold greater experimental efficiency than the traditional OVAT approach. Additionally, the use of DoE allowed us to glean new insights into the behavior of the CMRF of a number of arylstannane precursors. This information has guided our decision-making efforts while developing efficient reaction conditions that suit the unique process requirements of ^18^F PET tracer synthesis.

## Introduction

Positron emission tomography (PET) has become an important imaging technique that is used routinely in clinical practice and as a powerful biomedical research tool^1^. PET, as with other nuclear imaging modalities, relies on the appropriate use of well-designed radiotracers, molecules that are labelled with a positron emitting radionuclide and are designed to target and accumulate in specific organs, cells, diseased tissues, and/or biochemical pathways, providing physiological and molecular information about the subject^2^. The accessible and flexible design and radiosynthesis of novel tracers is a cornerstone of PET imaging as a preclinical research technique and the efficient and scalable development and production of new PET tracers is vital to the advancement of PET imaging as a clinically relevant tool^3^.

Of the many radioisotopes that can be readily produced with small medical cyclotrons, ^18^F has become particularly popular for medical imaging due to its almost ideal nuclear properties. Its decay mode (97% by positron emission), short positron range in tissue, high specific activity and practical 110-minute half-life have made it an attractive isotope for both clinical PET imaging and preclinical research and development^2,4^. However, in large part due to fluoride’s large hydration energy, basicity, and weak nucleophilicity, late stage radiofluorinations are synthetically challenging^5^. These reactions have, up until recently, been restricted to a relatively small subset of nucleophilic substitution reactions on aliphatic carbons (S_n_2) or electron-deficient aromatic rings (S_n_Ar). The limited number of synthetic tools available to radiochemists has in turn restricted the diversity and accessibility of new ^18^F radiotracers and has hence hindered their development^3^. Additionally, most clinical and pre-clinical radiosyntheses need to be carefully designed so that they can be performed in automated synthesis modules, which adds an additional layer of complexity when developing scalable and clinically relevant ^18^F tracer syntheses^2^.

**Figure 1:**
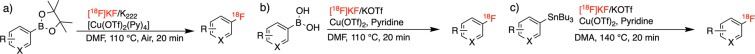
Recent copper-mediated nucleophilic radiofluorinations of electron-rich and electron-neutral (**a**) arylboronic esters by Tredwell *et al*., (**b**) arylboronic acids by Mossine *et al*., and (**c**) arylstannane precursors by Makaravage *et al*.^9–11^.

Recently however, new ^18^F labeling methodologies have been published that have begun to push the field forward, opening new avenues for radiotracer design and synthesis^2,5–8^. Seminal works published by the groups of Sanford, Gouverneur and Scott have provided unprecedented new synthetic tools for the late-stage radiolabeling of electron-rich and -neutral aromatic rings through the copper-mediated radiofluorinations (CMRF) of aryl boronic acids, aryl boronic esters and arylstannanes (Figure 1) ^9–13^. These reactions have been demonstrated through the synthesis of a number of clinically relevant tracers, and a number of groups, including our own, have begun to adopt these methodologies for the development of novel PET tracers^14,15^.

**Figure 2:**
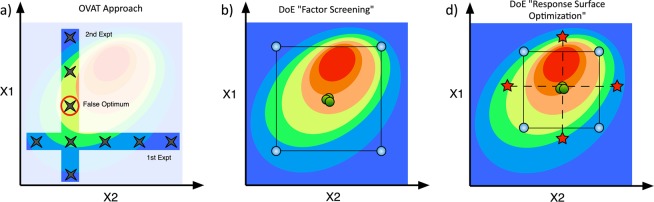
(**a**) The OVAT approach resolves reaction space one dimension at a time. The DoE approach builds a matrix of experimental runs to model a response surface across all reaction space. Different design types allow for (**b**) efficient factor screening studies or (**c**) more focused and detailed response surface optimization studies. The color grading represents the value of the true response (blue low, red high.) This figure has been recreated and modified with permission from the catalysisconsulting.co.uk website^35^.

However, many of these reactions have suffered from poor reproducibility and synthesis performance at larger scales. Works by a number of groups have identified the processing of the ^18^F (through QMA cartridge elution and azeotropic drying) as a critical step as the copper mediator is particularly sensitive the strong bases present in standard QMA eluents^14,15^. A number of efficient protocols to improve ^18^F QMA recovery rates and reaction conversions have thus been developed^16–20^. These general “unified” conditions, which include the popular “minimalist” ^18^F processing approach, have allowed CMRF chemistry to become a more frequently utilized tool for novel tracer development. However, in addition to the ^18^F processing method, CMRF reactions are themselves also complex, multicomponent processes and thus require the optimization of multiple nuanced, non-linear, and (as we will show) precursor specific experimental factors. The synthesis of almost every novel tracer (with the goal of automation) must undergo an extensive optimization process with respect to the reaction conditions, especially where new methodologies are utilized. This remains a crucial yet difficult, expensive, and often rate limiting step in the tracer/synthesis development pipeline.

The uptake of new radiochemical methodologies into routine use is heavily dependent on the reaction’s optimized operational simplicity, scalability, reliability, and efficiency in terms of both radiochemical conversion (%RCC) and byproduct formation (radiochemical purity and specific activity)^3^. Traditionally, these methodologies are optimized through the “one variable at a time” (OVAT) approach, which aims to hold all reaction variables (*X*_*i*_) constant while one is adjusted until a maximum %RCC or isolated radiochemical yield (%RCY) (response, *Y*_*i*_) is observed. This process is repeated until all factors suspected of effecting the response of interest have been optimized one by one (Fig. 2a) ^21^. This procedure is simple but laborious and time consuming, requiring many individual runs across an often-large number of parameters, many of which may have no significant contribution to the response. As this approach only looks at one factor at a time, it is unable to detect factor interactions, where the setting of one factor may affect the influence of another, and thus it often provides results that are difficult to interpret^22^. Additionally, the results of an OVAT study are dependent on the starting settings of the optimization process and as such, OVAT is prone to finding only local optima and may thus miss the true set of optimal conditions^23^.

An alternative to the OVAT approach is factorial experimental design or “Design of Experiments” (DoE), a systematic and statistical approach to process optimization that has been widely used by process engineers and chemists across a multitude of industries^24^. Unlike OVAT, DoE aims to explore, map and model the behavior of the response (or multiple responses) within a given reaction space (the combined ranges of all factors involved) across multiple factors simultaneously by varying all variables at once according to a predefined experimental matrix (Fig. 2b,c). DoE is thus able to provide a more detailed picture of the behavior of a particular process with experimental efficiency and is able to determine the contribution of each factor to the system, model the effect of each factor on the response, and resolve factor interactions. Even with low-resolution factor screening designs, where multiple factors maybe confounded, DoE aids in decision making and in the planning of further optimization studies^21,25^. As DoE data is analyzed statistically across a whole study (using multiple linear regression (MLR)) the error throughout the regression model can be estimated without the need for the multitude of replicate experiments (with the exception of replicate centerpoint experiments which are used to calculate the pure error) typically performed in OVAT studies, further increasing the experimental efficiency of the approach. Furthermore, the advent of user-friendly software packages, such as *Modde* and *JMP*, has helped to lower the barrier of entry of DoE for researchers with basic experience in statistical analysis^26^. In addition to those cited here, the numerous practical and scientific advantages of the DoE approach have been well outlined in a number of excellent reviews^21–27^.

DoE studies are usually conducted in sequential phases to answer specific scientific questions and there are a large number of different DoE designs that can be used in various situations to maximize the amount and quality of information obtained from the lowest number of experimental runs^25,27^. Typically, a DoE optimization will begin with a low resolution (highly confounded) fractional factorial screening design (Sup. Fig. 1a,b) in order to screen a large number of continuous (temperature, reagent stoichiometry, concentration, time, etc.) or discrete (atmosphere, solvent, reagent identity, etc.) variables that may affect the investigated response (%RCC, specific activity (SA), etc.) These “factor screening” (FS) experiments are designed to ascertain which factors have the largest influence on the response, give limited information on the presence of factor interactions and eliminate non-significant factors in as few runs as possible. They are thus usually not detailed enough to provide an accurate, predictive model of the system in question. Once the significant factors are identified, higher resolution response surface optimization (RSO) studies with a reduced subset of experimental factors can be constructed and performed if necessary (Sup. Fig. 1c,d). These designs usually contain more experimental points (per factor) and are intended to produce a detailed mathematical model of the process’s behavior.

DoE has been previously demonstrated as powerful tool for exploring and understanding new radiochemical methodologies^28,29^. In the context of copper-mediated radiosynthesis, DoE may provide a practical and efficient way to expedite the optimization process by increasing one’s understanding of the factors affecting the radiosynthesis of a new tracer at an early stage of its development. As DoE aims to maximize the information that can be obtained from a limited number of experimental runs, well-constructed DoE studies would save time, reduce the experimental resources (expensive cartridges, reagents and hot-cell/lead-castle time) devoted to the development of new methods and the optimization of synthesis protocols for new tracers, and would lower the exposure of researchers to harmful ionizing radiation.

The aim of the presented study was to assess the usefulness of a DoE approach to the study and optimization of the CMRFs of model arylstannanes as disclosed by Makaravage *et al*. and to glean to insights into the most important experimental factors that must be considered when attempting to optimize a tracer syntheses using this methodology^11^. This information was applied to an RSO DoE constructed to optimize the late-stage CMRF of 2-{(4-[^18^F]fluorophenyl)methoxy}pyrimidine-4-amine ([^18^F]*p*FBC), a novel tracer under development in our group that had previously suffered from poor synthesis performance and proved difficult to optimize through the conventional approach. Additionally, we used an RSO study to optimize the single step production of 4-[^18^F]fluorobenzyl alcohol ([^18^F]*p*BnOH), an ^18^F synthon of importance to a number of ongoing multistep radiosynthesis projects within our laboratory. In doing so, we highlight the use of DoE within the field of radiochemistry as a powerful tool to enhance radiochemical method development, expedite tracer synthesis optimization, and provide useful practical information about the process under investigation. This information could aid in general decision making when translating a radiosynthesis to an automated synthesis module, ultimately bringing it in line with current Good Manufacturing Practices (cGMP) for clinical production.

## Results and Discussion

### OVAT vs DoE: The advantage of better optimization routines

In order to assess the benefit of investigating the DoE approach for radiochemical process optimization, we studied the supplementary information of the original paper disclosing the CMRF of arylstannanes by Makaravage *et al*^11^. The authors investigated 8 non-discrete experimental factors, stating that each run was performed at least twice (n ≥ 2). Each factor was investigated across 3–6 different settings. Assuming n = 2 runs were performed for each setting, the authors therefore performed a minimum of 74 experimental runs (counted from the SI) to investigate the reaction’s behavior. Zarrad *et al*. later conducted a similar OVAT optimization study on a variation of this methodology that was based upon an improved QMA ^18^F processing method suitable for large-scale automated syntheses^16^. While their study successfully led to the development of a scalable and automatable procedure for the production of a number of PET tracers from aryltrialkylstannnes, it was also done with great experimental effort.

In contrast, a fractional factorial Resolution IV (RES IV) DoE study consisting of as few as 19 runs, could be performed to identify which factors had the largest influence on the response (Sup. Table 1). If, for example, 3 factors were identified as significant, a high-resolution response surface optimization experiment (consisting of only 17 runs) could then be carried out to estimate a more detailed map of the experimental space. Thus, the DoE approach (across both FS and RSO studies) would, if valid, provide a more comprehensive model of the process in just 36 (vs 74) runs. This marked, potential improvement in experimental efficiency, prompted us to further investigate DoE as a tool for radiochemical optimization.

### Factor screening of the CMRF of arylstannanes

**Figure 3:**
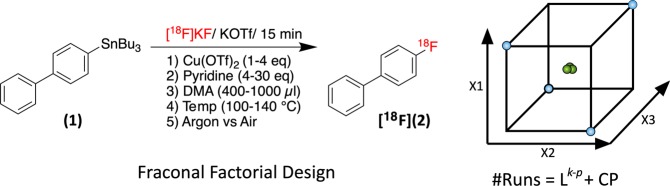
The investigated factors and their ranges for the fractional factorial factor screening of the model synthesis 4-[^18^F]fluorobiphenyl (**[**^**18**^**F]2**) from 4-tributyltinbiphenyl (**1**). In a fractional factorial design, experimental points are arranged at the corner of a K-dimensional hypercube. p is the total number of generators used to form the array (1/K^p^ is the fraction of the total number of runs from the full factorial experiment (all vertices of a hypercube). Center points (CP, shown in Green) are repeated experiments carried out at the center of the hypercube to estimate reproducibility and measure curvature in the response surface.

In order to identify the factors that had the most significant effects on the reaction outcome, a factor screening Resolution V + (RES V + ) fractional factorial design (capable of resolving main effects, 2 factor interactions, and revealing the presence of curvature in the model) was constructed using *Modde Go 12* (Umetrics). 4-Tributylstannylbiphenyl (**1**) was chosen as a model substrate due its availability, the low volatility of the product 4-[^18^F]fluorobiphenyl (**[**^**18**^**F]2**) on TLC plates, and its prevalence in the literature as a standard model compound for radiofluorination method development (Fig. 3)^11^. The precursor amount was set at 2 mg (4.5 µmol) across all runs. Five factors, namely: Reaction solvent volume (DMA vol: 400–1000 µl DMA), temperature (Temp: 100–140 °C), copper triflate loading (Cu(OTf)_2_: 1–4 eq relative to substrate), pyridine loading (Pyridine: 4–30 eq), and atmosphere (Atm: argon vs air) were identified in pilot experiments and though literature consultation as factors of interest. A number of previous studies, including that reported by Zarrad *et al*., have reported enhanced yields when these reactions are performed in air^9,15,16,19^. The effect of using argon or air on the %RCC was however difficult to compare and quantify during pilot experiments and thus it was included as a qualitative factor (argon or air) in the factor screening DoE. Time was not investigated as a factor as i) time is related to temperature in most chemical process and ii) given the short half-life time ^18^F, it is more desirable to set a reaction duration of < 30 min. The radiochemical conversion of the reaction (%RCC) was chosen as the response (*Y*_*%RCC*_), as it can be quickly and accurately measured by radioTLC.

The fractional factorial experimental design entailed a total of 24 experimental runs composed of 16 experimental points with 8 center point experiments. Due to the practical constraints of processing and using radiofluoride, the factor screening DoE (and future RSO DoE studies) needed to be run over multiple days. It was decided, given the time required to perform each reaction, that 6 experiments per day was optimal. To account for uncontrollable factors brought about through day-to-day variances in radiofluoride quality and quantity, QMA cartridge variations, and variations in QMA eluent, the experiments were arranged into 4 blocks of 6 runs. Each block, which contained two replicate center points to assess reproducibility, would be included into the model as blocking factors to account for variations in day-to-day uncontrollable factors.

^18^F trapped on a QMA cartridge (preconditioned with NaHCO_3_) was eluted with the QMA eluent as published by Makaravage *et al*. and was divided among the 6 reaction vials in 80 ul aliquots. The limitations of this “aliquot” method have been well documented in the literature^15^. The lower base/salt content present in smaller aliquot volumes of QMA eluent has less of a negative effect on %RCC than if a full QMA eluent “batch” is used. As such, %RCC values obtained via the aliquot method are often not representative of the %RCC obtained from large-scale batch elutions of ^18^F with the same QMA eluent. However, despite this limitation, we chose to aliquot the ^18^F into each reaction as this would better allow us to measure and account for variances in each QMA cartridge elution from day-to-day (between blocks) and would also ensure that the QMA eluent content present in each reaction vial after azeotropic drying would be reasonably stable within each block. The minimization of large sources of experimental error was of paramount important to the construction of an accurate DoE model.

**Figure 4:**
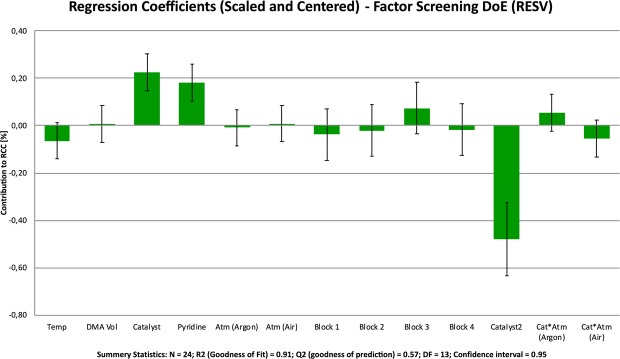
The scaled and centered regression factors calculated from the results of fractional factorial factor screening DoE. Large regression coefficients represent factors with large contributions to the response (%RCC). A positive number denoted a positive influence on the response. A negative number denotes a diminishing effect on the response. To fit an accurate model, non-significant terms would need to be eliminated, but for the purposes of factor screening, these non-significant terms are shown here. If a factor’s regression coefficient is smaller than the associated errors bars it is probable (at the 95% confidence interval) that that factor is not significant.

After performing each run, the experimental results were analyzed using *Modde Go 12*. To obtain a normal distribution of the data, the %RCC data set was transformed to the log(10), fitted to a model using multiple linear regression (MLR), and checked for outliers and model quality. The output summary statistics suggested the model to be good enough for the purposes of factor screening (R^2^ = 0.91 (goodness of fit), Q^2^ = 0.57 (goodness of model prediction). The normal coefficients of each term in the model were used to gauge the significance of the contribution of the corresponding factors to the response (p = 0.05) (Fig. 4). The model suggested that both temperature (Temp) and total DMA volume (DMA Vol: reaction volume/concentration) were non-significant factors over the investigated ranges. Catalyst loading, (Cu(OTf)_2_) and ligand loading (Pyridine) were determined to be significant factors. The model also suggested the presence of curvature in the response surface, but due factor confounding inherent in the (RES V) experimental design, a more detailed RSO experimental would need to be conducted to determine which quadratic terms would be required to fit an accurate model. The presence of missing quadratic terms in the linear factor screening model could explain the low Q^2^ term in the model fit statistics. Additionally, no significant differences between the experimental blocks (Block 1–4) were observed, suggesting the experimental protocol to be stable from day-to-day.

The factor screening DoE also suggested that, when using stoichiometric quantities of Cu(OTf)_2_ (1–4 eq), the choice of atmosphere (Atm (argon/air)) was not a significant factor and the presence of atmospheric oxygen does not significantly enhance the reaction over the ranges investigated. Interestingly however, a non-significant factor interaction between catalyst loading an atmosphere was detected. At high Cu(OTf)_2_ loadings, an argon atmosphere is slightly preferred, while at low Cu(OTf)_2_ loadings, an air atmosphere is beneficial. While its insignificance warrants that it is excluded from further experimental designs and models, the trend suggested by this interaction fits in line with the current understanding of the oxidation cycle of the Chan-lam coupling^30^. When catalytic quantities of Cu(II)(OTf)_2_ are used, an oxidative atmosphere (Air) is required to activate catalytic complex to a Cu(III) species and to regenerate the catalyst after it undergoes reductive elimination. When larger amounts of Cu(II)(OTf)_2_ are used, the reaction can be performed under argon as the oxidation of the inactive Cu(II) complex to the active Cu(III) complex is mediated by free Cu(II) through a single electron transfer^31^. An important conclusion from this result is that this CMRF can be performed in automated synthesizers using inert carrier gases; operating these reactions under air is not a requirement when stoichiometric loadings of Cu(OTf)_2_ are used, as was originally suggested by Makaravage *et al*.^11^. Most routine radiosynthesis modules are setup and optimized to operate using an inert carrier gas such as nitrogen, argon or helium. While it is possible to setup and operate many synthesis modules using compressed air, it can be inconvenient to modify/change/switch established routine (or GMP) syntheses and synthesis modules to operate with air.

### Response surface optimization of [^18^F]pFBC

**Figure 5:**
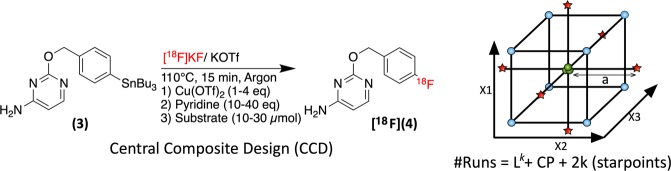
The investigated factors and their ranges for the orthogonal central composite design RSO of [^18^F]pFBC (**[**^**18**^**F]4**). Starpoint distance a is scaled in order to ensure orthogonality throughout the experimental matrix. An orthogonal central composite design (CCO) has a distance “a” scaled so as to ensure orthogonality in the experimental matrix.

[^18^F]*p*FBC (**[**^**18**^**F]4**), produced from precursor (**3**), is novel tracer under development in our laboratory that had shown poor synthesis performance and reliability (Fig. 5). Our efforts to optimize its synthesis iteratively through the OVAT approach in conjunction with previously published optimization data had given inconsistent and confusing results^11,16^. Thus, having identified and eliminated reaction solvent volume, temperature, atmosphere, and day-to-day uncontrollable factors as non-significant factors, a more detailed orthogonal central composite design (CCO) RSO study was constructed to optimize the radiosynthesis of this tracer. Cu(OTf)_2_ loading (1–4 eq), pyridine loading (10–40 eq), and precursor loading (10–30 µmol) were chosen as factors for investigation. The reaction volume was kept constant across all runs at 700 µl and each run was performed at 110 °C for 15 min.

**Figure 6:**
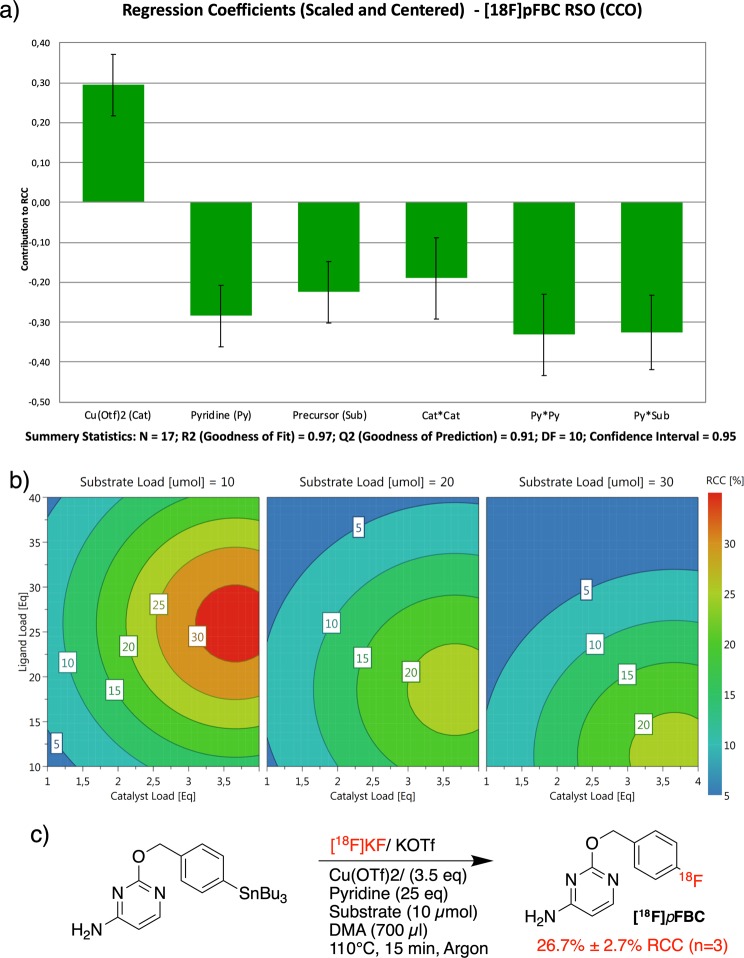
(**a**) The scaled and centered regression factors calculated from the results of the RSO (CCO) (**a**) 4D plot output from Modde Go 12. Pyridine (ligand) and catalyst loadings are plotted on the vertical and horizontal axis respectively. The three windows, from right to left, represent an increasing amount of substrate (10–30 µmol). (**c**) Reaction conditions and radiochemical conversions of the optimized CMRF synthesis of [^18^F]*p*FBC.

The CCO design, a type of central composite design (CCD), was chosen due to its ability to estimate second order response surfaces and resolve quadratic terms in the response surface model. The CCO design consisted of a total of 17 runs: 8 factorial points, 3 center points and 6 orthogonally scaled star points (Fig. 5). The 17 runs were again carried out using 80 µl aliquots of ^18^F in accordance with the general procedure described in the supplementary information. The data was modeled using MLR and analyzed in *Modde Go 12*. All three main factors were found to be significant, and the experiment also resolved quadratic behaviors for both catalyst and pyridine loading factors (Fig. 6a). Additionally, a factor interaction between pyridine and substrate loading was resolved and included in the model. The summary of fit statistics gave R^2^ and Q^2^ to be 0.97 and 0.91 respectively, indicating a valid and predictive model. All three main factors had significant effects on the response. Strong quadratic behaviors were found for both Cu(OTf)_2_ loading and pyridine factors, and a strong negative factor interaction was detected between the equivalents of pyridine and the amount of substrate used (higher amounts of pyridine are needed for lower amounts of precursor.) Plotting the response surface across the investigated ranges suggested that the optimal set of conditions consisted of 3.5 equivalents of catalyst and 25 equivalents of pyridine (a ratio≈1:7) at a 10 µmol substrate load (Fig. 6b). Thus, three validation runs were performed using larger 180 µl aliquots of the QMA solution (400–500 MBq) under the optimized conditions (Fig. 6c). These three runs gave respective %RCCs of 24.9%, 25.3%, and 29.8% (26.7 ± 2.7%RCC (n = 3)), demonstrating the robustness of these conditions and giving the highest %RCCs obtained for [^18^F]*p*FBC thus far, using this reaction.

### Response surface optimization of the synthesis of [^18^F]4-fluorobenzyl alcohol ([^18^F]pFBnOH)

**Figure 7:**
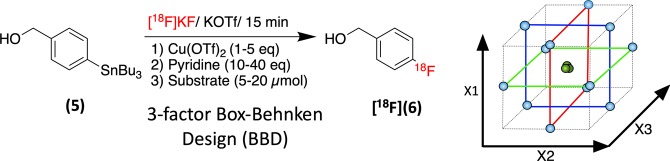
The investigated factors and their ranges of the Box Behnken response surface optimization design for the synthesis of [^18^F]*p*FBnOH (**[**^**18**^**F]6**) from p-tributyltin-benzyl alcohol (**5**). The BBD arranges the experimental points on the edges of the reaction space cube and can be thought of as a combination of three 2D full factorial designs (performed at 90° to each other) with shared center points.

The synthesis [^18^F]*p*FBnOH **[**^**18**^**F]6**, an important radiochemical building block, has also been of interest to a number of projects within our laboratory. **[**^**18**^**F]6** has been previously synthesized in two steps via the nucleophilic aromatic substitution of 4-formyl-*N,N,N*-trimethylanilinium triflate and the subsequent reduction of the resulting 4-[^18^F]fluorobenzaldehyde to **[**^**18**^**F]6**^32,33^. In our hands the reduction step using NaBH_4_ resulted in a significant loss of the product **[**^**18**^**F]6** and we thus chose to investigate the CMRF of 4-tributyltinbenzyl alcohol **5** as a possible single-step alternative route to **[**^**18**^**F]6** (Fig. 7). **[**^**18**^**F]6** could be reasonably purified via solid-phase extraction before use in a second synthesis step (these results will be published in due course.) Using the information obtained from our previous DoE studies, an RSO experiment was constructed to optimize the synthesis of **[**^**18**^**F]6** using a Box Behnken Design (BBD) (Fig. 7). The BBD requires slightly fewer runs than an equivalent CCD and also avoids experimental runs with combined extremes of the experimental factors. The three factor Box-Behnken design featured a total of 15 runs (12 experimental points with 3 center points). Again, substrate loading (5–25 µmol), catalyst loading (1–4 eq) and pyridine loading (5–30 eq) were chosen as factors for investigation. The reaction volume was again kept constant across all runs at 700 µl and the reactions were each performed according to the general procedure at 110 °C for 20 min.

**Figure 8:**
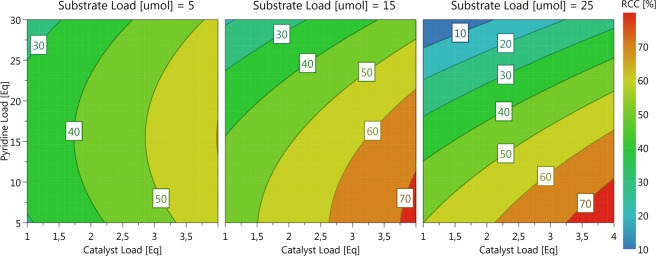
The response surface output from the Box Behnken response surface optimization of **[**^**18**^**F]6**.

Fitting the data using MLR in *MODDE Go 12* gave summary of fit statistics that suggested a valid model (R^2^ = 0.97 and Q^2^ = 0.86). Catalyst loading and pyridine loading were found to be significant factors, with pyridine demonstrating a quadratic behavior. In this case, precursor loading was not found to be a significant factor over the investigated range. Plotting the response surface suggested that the optimum reaction conditions featured a high catalyst load and a low pyridine load with a higher substrate load being slightly (non-significantly) beneficial (Fig. 8). Again, validation runs with larger 180 µl ^18^F aliquots were performed as before using a substrate loading of 25 µmol, 4 equivalents of Cu(OTf)_2_ and 5 equivalents of pyridine in 700 µl of DMA. The outcome afforded **[**^**18**^**F]6** with a %RCC of 58 ± 5.3% (n = 4) in a single step. While these results were less than the those predicted by the response surface model, they again provided the product with greater efficiency than previously obtained in our hands using the general fluoride processing and reaction conditions published by Makaravage *et al*.

In this case, deviations from the predicted model may be due to factors such as the amount of carbonate base present in the in larger volumes of QMA eluent solution (as discussed above), effects from as of yet unidentified controllable or non-controllable factors specific to this reaction, and/or model/data inaccuracies obtained through either random or systematic experimental error. Nonetheless, this easily automatable procedure mitigated the product losses sustained using the previously published 2-step synthesis approach^32^.

These data were used to successfully guide the automation of [^18^F]*p*FBC as well as [^18^F]*p*FBnOH (as part of larger multistep radiosynthesis project) on an Elixys Flex/Chem synthesis module (Sofie Biosciences, USA). These automated syntheses will be reported in due course as part of a larger tracer development study. It must however be noted that the radiochemical yields of the automated synthesis were, as expected, significantly lower than those predicted by the response surface model. This is in all likelihood due to the differences in single “batch” ^18^F processing methods that were used in the automated synthesis versus the “aliquoted” ^18^F processing that was used to carry out the DoE experiments. Although large scale radiosyntheses were nonetheless useful for imaging studies, this remains a significant limitation of the presented DoE studies. However, we suspect that the results of our reaction optimization can viewed independently from the known issues associated with fluoride processing, and we are currently working to confirm this hypothesis. As such, we believe that the application of improved fluoride processing techniques, such as the “minimalist” approach to fluoride processing, may help to drastically improve the large-scale performance of our optimized copper-mediated radiofluorination conditions and we are currently working to implement these methods into our workflow.

Comparing our factor screening and response surface models with the results obtained from the previous OVAT optimization studies by Makaravage *et al*. and Zarrad *et al*. reveals remarkably similar trends where the models are comparable (absolute %RCC values differ considerably due to the difference in the ^18^F processing methods used.) For example, substrate load and copper triflate loading both show quadratic behaviors and their optima are reasonably well aligned with the analogous regions in our response surface models, despite the large differences in ^18^F processing method. This lends weight to our hypothesis that the experimental factors affecting the reaction can be modeled separately from the ^18^F processing conditions; i.e., there is no (or only a weak) factor interaction between the ^18^F processing conditions used and reaction parameters we have investigated in this study; however, this still requires further investigation and will be reported on in due course. The multiparametric response surfaces provided by the DoE studies presented here also highlight the fact that much more information about a process can be obtained from fewer experiments if the DoE approach is appropriately applied.

Comparison of the two response surface models for [^18^F]*p*FBC and [^18^F]*p*FBnOH shows that the later requires a lower quantity of pyridine and a higher substrate concentration for optimal radiolabeling, while the synthesis of [^18^F]*p*FBC benefits from a lower substrate concentration and higher pyridine load. This suggests that the nature of the substrate is a major factor when developing optimal CMRF reaction conditions. The presence of some heterocycles has been previously noted to have marked deleterious effects on %RCC, likely due to the formation of unreactive substrate/catalyst species. Taylor *et al*. examined the effects of various substrates on the analogous CMRF of boronic acid esters by performing reactions with a model substrate, while holding the reaction conditions constant and doping the reactions with various heterocycles and other common moieties often found in drug-like molecules^34^. From their results, they were able to construct a database of heterocyclic moieties that are compatible with their radiofluorination conditions that could be used to plan and “de-risk” future radiosyntheses. Our data suggests that, in certain cases, a detailed understanding of the process and careful optimization of important experimental factors could (to some degree) offset these deleterious effects, thus saving time by reducing the need to design complex multistep synthesis routes around problematic moieties in the candidate precursor. In combination with a database of problematic moieties (such as that published by Taylor *et al*.), well-designed DoE studies could aid in the establishment of useable radiofluorination protocols early on in a tracer’s development and thus expediate its passage from conception to its first preclinical studies. Scientist can then quickly decide if the tracer is biologically interesting and if further optimization or development of an improved synthetic strategy for GMP production is indeed warranted.

## Conclusion

The work presented here highlights the benefit of using the DoE approach to aid in the development of new radiochemical methodologies as well as PET tracer development and production. The systematic use of the DoE approach streamlines the optimization process, saving time and resources while providing multiparametric information that can be used to guide decision-making early on during a tracer’s development. While we have specifically investigated the use of DoE for optimizing the copper-mediated radiofluorination of arylstannanes as proof of principle, it is important to note that DoE can be applied to any complex optimization problem. The availability of a number of easy to use DoE software packages (such as *Modde Go 12* and *JMP*) has allowed us to apply DoE to the synthesis optimization of a number of novel tracers under development and we are currently applying the presented DoE data and the general DoE approach to expedite the delivery of a number of biologically interesting tracers to imaging scientists within our group. We have also begun to explore the use of DoE as a research tool to guide reaction development and aid in the establishment of new radiochemical methodologies within our laboratory. We hope that DoE will become a more widely used tool that will help bring new radiochemical methods into clinical and preclinical relevancy and will in turn help expand the chemical diversity of new ^18^F labelled tracers.

## Methods

The synthesis procedures and characterization data of all precursor and non-radioactive standard compounds can be found the supplementary information attached to this paper along with the DoE design worksheets and regression model statistics.

### General radiochemistry

As a general procedure for all radiochemical experiments, [^18^F]fluoride in water was obtained from a cyclotron (GE PETtrace 800) target wash and was trapped on a QMA cartridge (QMA Light Carb, Waters; preconditioned sequentially with 1 M NaHCO_3_ (10 ml), air (10 ml), Water (10 ml), and air (10 ml)), and eluted with a QMA eluent solution (K_2_CO_3_ 50 µg, KOTf 10 mg in H_2_O 550 µl.) (published by Makaravage *et al*.) To ensure consistency in the potassium [^18^F]fluoride and potassium triflate content introduced from the QMA eluent, the eluted radiofluoride was aliquoted (80 µl) into 6 × 5 ml Wheaton (V-vials, oven dried) reactors (200–300 MBq) and each reactor was separately azeotropically dried at 110 °C with acetonitrile (3 × 1 ml) under a stream of argon gas. (As opposed to drying a single batch and aliquoting the poorly soluble [^18^F]KF thereafter.) The reaction mixtures required by the DoE worksheet table were formulated from stock solutions of the required reagents in DMA (1 mg / 10 µl) and diluted with DMA to the required reaction volume. Reactions run under argon were purged with a stream of argon gas for 20 seconds. Reactions run under air were purged with air in similar fashion. The reactions were set to run at the required temperature for the specified time, after which they were quenched with 1 ml of water to solubilize the remaining fluoride. Samples of each reaction were taken for analysis.

### Reaction analysis

RadioTLC was used to determine the relative incorporation of radiofluoride by the substrate and both product and by-product signals were quantified in order to determine %RCC. HPLC analysis was performed on representative samples over the course of the DoE studies to ensure compound identity.

### Experimental design and analysis

All DoE studies were designed using the DoE software package *Modde Go 12* (*Umetrics*). After the factors and responses of interest were defined, an appropriate design type was selected, and a DoE experimental worksheet table was generated. All experiments were performed in randomized order. After the %RCC data was collected, the data was modelled using MLR, checked for outliers and model quality, after which is could be used for the purposes of factor screening or response surface optimization.

## Supplementary information

Supplementary Information
