# Supplementary material for: A Design of Experiments (DoE) Approach Accelerates the Optimization of Copper-Mediated 18F-Fluorination Reactions of Arylstannanes
Source: Sci Rep. 2019 Aug 6;9:11370. doi: 10.1038/s41598-019-47846-6 (PMC6684620; doi:10.1038/s41598-019-47846-6)
Supplement: Supplementary file 1 — Supplementary Information [file 41598_2019_47846_MOESM1_ESM.pdf]

Supplementary Information:

A Design of Experiments (DoE) Approach Accelerates the Optimization of Copper-Mediated ^{18}F-Fluorination Reactions of Arylstannanes.

Gregory D. Bowden[1], Bernd J. Pichler[1,2], Andreas Maurer[1,2,*]

[1]Werner Siemens Imaging Center, Department of Preclinical Imaging and Radiopharmacy, Eberhard Karls University, Tübingen, Germany

[2]iFIT-Cluster of Excellence, Eberhard Karls University, Tuebingen, Germany

*Correspondence to andreas.maurer@med.uni-tuebingen.de

Table of Contents

1. *Organic Synthesis* 3

1.1. General Materials and Methods: 3

1.2. 4-(tributylstannyl)biphenyl (1)[1] 3

1.3. 2-((4-(tributylstannyl)phenyl)methoxy)pyrimidin-4-amine (3) 4

1.4. 2-((4-[^{18}F]fluorophenyl)methoxy)pyrimidin-4-amine (4) 4

1.5. 4-(tributylstannyl)benzyl alcohol (5)[2] 5

2. *NMR Spectra:* 6

2.1. 4-(tributylstannyl)biphenyl (1) 6

2.2. 2-((4-(tributylstannyl)phenyl)methoxy)pyrimidin-4-amine (3) 7

2.3. 2-((4-fluorophenyl)methoxy)pyrimidin-4-amine (4) 8

2.4. 4-(tributylstannyl)benzyl alcohol (5) 9

3. *Radiochemistry:* 10

3.1. General Procedure for DoE radiochemistry experiments 10

3.2. Analytical HPLC Conditions: 10

3.3. Representative Radiochemical Analysis: 4- [^{18}F]fluorobiphenyl 11

3.4. Representative Radiochemical Analysis: 2-((4-[^{18}F]fluorophenyl)methoxy)pyrimidin-4-amine ([^{18}F]*p*FBC) 12

3.5. Representative Radiochemical Analysis: 4-[^{18}F]fluorobenzyl alcohol ([^{18}F]*p*BnOH) 13

4. *DoE:* 14

4.1. Factor Screening on the CMRF of a Model Arylstannane: 16

4.2. Response Surface Optimization For the synthesis of [^{18}F]pFBC 17

4.3. Response Surface Optimization or the Synthesis of [^{18}F]FBnOH: 19

5. *References* 21

1. Organic Synthesis

1.1. General Materials and Methods:

All reagents and solvents were purchased from Merck, Sigma-Aldrich, Tokyo Chemical Industry (TCI), or Karl Roth and used without further purification unless otherwise stated. ^{1}H and ^{13}C NMR spectra were obtained from compounds dissolved in $CDCl_3$ at 300 K using a Bruker AV 600 (^{1}H: 600.13 MHz and ^{11}C: 150.03 MHz) spectrometer, respectively. Chemical shifts were referenced to residual $CHCl_3$ (δ_H = 7.28), $CDCl_3$ (δ_C = 77.00). TLC was carried out on 0.25 mm thick precoated Merck plates, silica gel 60 F_{254}. Flash (column) chromatography was performed with silica gel 60 (230-400 mesh). Spots were visualized by UV and/or dipping the plate into a solution of molybdophosphoric acid in ethanol followed by heating with a heat gun. Analytical HPLC-Ms data was collected using an Agilent HPLC (1200 series) coupled to quadrupole MS detector (Agilent, 6120 series, ESI mode) with under the following conditions. Column: Luna 5µm C-18 100 Å, 50 x 2 mm; Solvent A: H_2O + TFA (0.1%); Solvent B: acetonitrile; Gradient: 0 min (0% B), 7.6 min (100% B), 7.8 min (100% B), 8.30 min (0% B), 12.0 min (0% B).

1.2. 4-(tributylstannyl)biphenyl (1)[1]

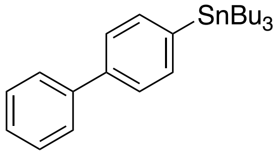

The synthesis of 4-(tributylstannyl)biphenyl was adapted from the published procedure.[1] A two-neck reaction flask fitted with a rubber septum was purged with argon and charged with 4-bromobiphenyl (500 mg, 2.15 mmol) in dry diethyl ether (20 ml). The reaction vessel was cooled to -78 °C using an acetone/dry ice bath under a stream of argon, after which *n*-BuLi in hexane (2.5 M, 950 µl, 2.36 mmol) was added through the septum to the stirring solution. The reaction mixture was allowed to warm to room temperature and left to stir for 1 hour, after which it was again cooled to -78 °C. Tributyltin chloride (780 µl, 2.36 mmol) was then added to the organolithium derivative in dropwise fashion and the reaction was then warmed to room temperature and left to stir overnight. The reaction was quenched with sat. ammonium chloride (10 ml), diluted with Et_2O and poured into a separating funnel containing water (30 ml) and Et_2O (30 ml). The product was then extracted from the aqueous phase with Et_2O (3 x 30 ml), the organic fractions were collected and dried with magnesium sulfate after which the solvent was removed *in vacuo.* The remaining residue was purified by silica chromatography (1% - 10% ethyl acetate in hexane) to afford the product as a colorless oil (798 mg, 84%). NMR data were found to be identical to previously published spectra.

^{1}H NMR (600 MHz, $CDCl_3$): δ 7.64 – 7.59 (m, 2H, Ar), 7.59 – 7.50 (m, 4H, Ar), 7.48 – 7.40 (m, 2H, Ar), 7.37 – 7.32 (m, 1H, Ar), 1.67 – 1.48 (m, 6H, $SnBu_3$-C$\underline{H}_2$), 1.36 (m, 6H, $SnBu_3$-C$\underline{H}_2$), 1.16 – 1.02 (m, 6H, $SnBu_3$-C$\underline{H}_2$),

0.91 (t, *J* = 7.3 Hz, 9H, SnBu$_3$-CH$_3$); ^{13}C NMR (151 MHz, CDCl$_3$): δ 141.5 (Ar), 141.0 (Ar), 140.9 (Ar), 137.0 (Ar), 128.9 (Ar), 127.3 (Ar), 127.3 (Ar), 126.8 (Ar), 29.3 (SnBu$_3$), 27.6 (SnBu$_3$), 13.8 (SnBu$_3$), 9.8 (SnBu$_3$).

1.3. 2-((4-(tributylstannyl)phenyl)methoxy)pyrimidin-4-amine (3)

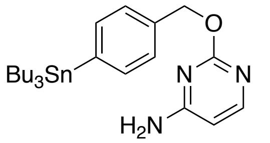

To a suspension of sodium hydride (60% in mineral oil, 308 mg, 7.70 mmol) in DMSO (5 ml) was added a solution of the alcohol **5** (1.68 mg, 4.24 mmol) in DMSO (2 ml) in dropwise fashion over a period of 2 minutes. The resulting solution was left to stir vigorously for 1 hour at room temperature after which 2-chloro-4-aminopyrimidine (500 mg, 3.85 mmol) was added in small portions under a gentle flow of argon. The reaction mixture was warmed to 110 °C for 2 hours and monitored by TLC. When the reaction was deemed to be complete, it was quenched by pouring the reaction mixture into a separating funnel containing aqueous saturated ammonium chloride (250 ml). The product was then extracted with ethyl acetate (3 X 80 ml), dried with magnesium sulfate, evaporated in vacuo, purified via column chromatography (10% - 80% ethyl acetate in hexane) to afford the desired compound as a thick oil (1.12 g, 63%).

^{1}H NMR (600 MHz, CDCl$_3$): δ 8.01 (d, *J* = 5.8 Hz, 1H, ArCH), 7.51 – 7.34 (m, 4H, Ar-SnBu3), 6.14 (d, *J* = 5.8 Hz, 1H, ArCH), 5.35 (s, 2H, ArCH$_2$O), 5.15 (s, 2H, NH$_2$), 1.53 (m, 6H, SnBu$_3$-CH$_2$), 1.38 – 1.27 (m, 6H, SnBu$_3$-CH$_2$), 1.15 – 0.95 (m, 6H, SnBu$_3$-CH$_2$), 0.88 (t, *J* = 7.3 Hz, 9H, SnBu$_3$-CH$_3$); ^{13}C NMR (151 MHz, CDCl$_3$): δ 165.0 (NCON), 164.8 (Ar), 156.8(Ar), 141.7 (Ar), 136.6 (Ar), 136.4 (Ar), 127.5 (Ar), 99.7 (Ar), 68.9 (Ar-CH$_2$-Ar), 29.2 (SnBu$_3$), 27.5 (SnBu$_3$), 13.8 (SnBu$_3$), 9.7 (SnBu$_3$); HPLC: (rt = 10.2 min); HPLC-MS (ESI) (m/z): [M]$^+$ calcd. for $C_{23}H_{37}N_3OSn$, 491.20; found [M+H]$^+$, 492.1.

1.4. 2-((4-[^{18}F]fluorophenyl)methoxy)pyrimidin-4-amine (4)

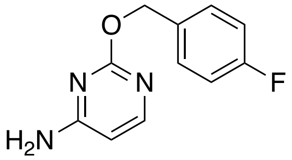

To an argon purged reaction vessel contained a cooled (0°C) suspension of sodium hydride (60% in mineral oil, 70 mg, 1.731 mmol) in DMF (2 ml) was added a solution of 4-fluorobenzyl alcohol (175 mg, 1.385 mmol) in DMF (1 ml) in dropwise fashion. The resulting solution was allowed to warm to room temperature for 1 hour after which it was again cooled to 0°C in an ice/water bath. 2-chloro-4-aminopyrimidine (150 mg, 1.154 mmol) was then added to the solution in small portions under a gentle stream of argon. The reaction mixture was then heated at 100°C for 6 hours after which it was allowed to cool to room temperature and left to stir overnight. The reaction was quenched with a saturated solution of NH_4Cl and poured into a separation funnel

containing NH_4Cl (sat.) and ethyl acetate (50 ml). The compound was extracted with a further portions of ethyl acetate (50 ml), the organic fractions were pooled and dried with magnesium sulfate. After concentrating the residue onto silica gel, the product was purified via column chromatography (15% - 80% ethyl acetate in hexane) to afford the desired product as a white crystalline solid (235 mg, 93%).

^{1}H NMR (600 MHz, DMSO-d_6): δ 7.9 (d, J = 5.7 Hz, 1H, $\underline{H}$C=CH), 7.5 – 7.4 (m, 2H, Ph), 7.2 – 7.1 (m, 2H, Ph), 6.9 (s, 2H, $N\underline{H}_2$), 6.1 (d, J = 5.7 Hz, 1H, HC=C$\underline{H}$), 5.2 (s, 2H, $OC\underline{H}_2Ph$); ^{13}C NMR (151 MHz, DMSO-d_6) δ = 165.4 (NCN), 164.5 ($\underline{C}N_2$), 161.6 (d, J=243.5, $(CH)_2\underline{C}F$), 156.2 (H$\underline{C}$=CH), 133.7(Ph), 129.9 (d, J=8.3, $(\underline{C}H)_2(CH)_2CF$), 115.0 (d, J=21.2, $(CH)_2(\underline{C}H)_2CF$), 99.6 (HC=$\underline{C}$H), 66.4($O\underline{C}H_2Ph$); HPLC: (rt = 4.34 min); HPLC-MS (ESI) (m/z): $[M]^+$ calcd. for $C_{11}H_{10}FNO$, 219.08; found $[M+H]^+$, 220.1.

1.5. 4-(tributylstannyl)benzyl alcohol (5)[2]

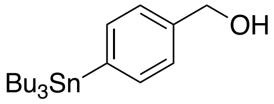

The synthesis of 4-(tributylstannyl)benzyl alcohol was adapted from the published procedure.[2] 4-bromobenzyl alcohol (2.00 g, 15.80 mmol) was dissolved in THF (80 ml) under a stream of argon in a two-neck Schlenk flask fitted with two equalizing dropping funnels. The reaction vessel was cooled to -78 °C using an acetone/dry ice bath under a stream of argon, after which *n*-BuLi in hexane (2.5 M, 12.72 ml, 31.80 mmol) was added dropwise via the first dropping funnel. The reaction was stirred at -78 °C for 1.5 hours under positive pressure of argon, after which tributyltin chloride was added dropwise through the second dropping funnel over a period of 45 min. The reaction was left to stir at -78°C under positive pressure of argon for 2 hours. It was then allowed to warm to room temperature and left to stir over night, after which it was quenched with saturated ammonium chloride solution (10 ml), diluted with Et_2O (20 ml), and transferred to a separating funnel containing saturated ammonium chloride (90 ml). The product was extracted with Et_2O (3 x 100 ml), the organic fractions were washed with brine (50 ml), dried with magnesium sulfate, and evaporated *in vacuo*. The resulting residue was purified by silica chromatography (5% - 20% ethyl acetate in hexane) to afford the product as clear oil (3.52 g, 56%). NMR data were found to be identical to previously published spectra.

^{1}H NMR (600 MHz, $CDCl_3$): δ 7.53 – 7.41 (m, 2H, Ar), 7.37 – 7.30 (m, 2H, Ar), 4.68 (s, 2H, $ArC\underline{H}_2OH$), 1.54 (m, 6H, SnBu3-C$\underline{H}_2$), 1.33 (m, 6H, $SnBu_3$-C$\underline{H}_2$), 1.13 – 0.98 (m, 6H, $SnBu_3$-C$\underline{H}_2$), 0.89 (t, J = 7.4, Hz, 9H, SnBu3-C$\underline{H}_3$); ^{13}C NMR (151 MHz, $CDCl_3$): δ 141.5 (Ar), 140.6 (Ar), 136.9 (Ar), 126.8 (Ar), 65.6 (Ar-$\underline{C}H_2$), 29.2 ($SnBu_3$), 27.5 ($SnBu_3$), 13.8 ($SnBu_3$), 9.7 ($SnBu_3$).

2. NMR Spectra:

2.1. 4-(tributylstannyl)biphenyl (1)

2.1.1. 1H NMR:

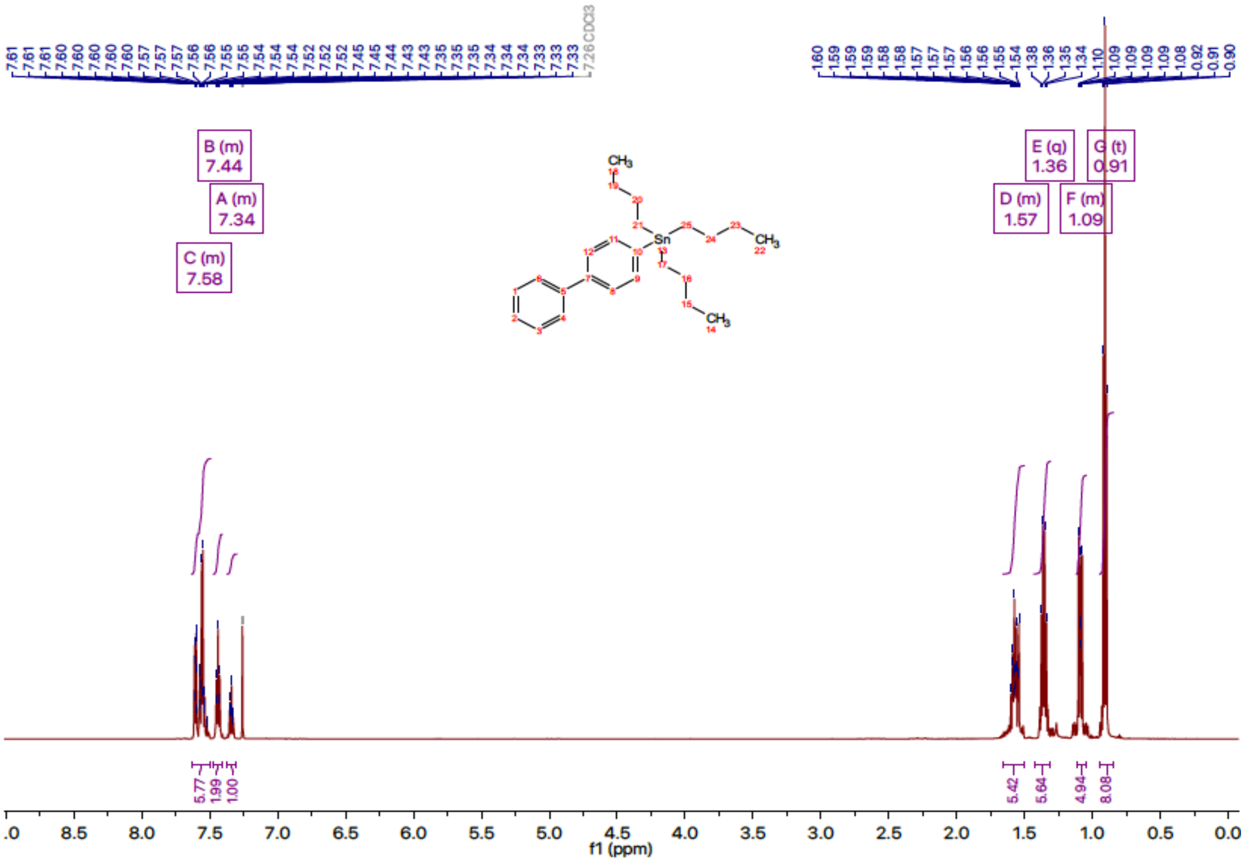

2.1.2. 13C NMR:

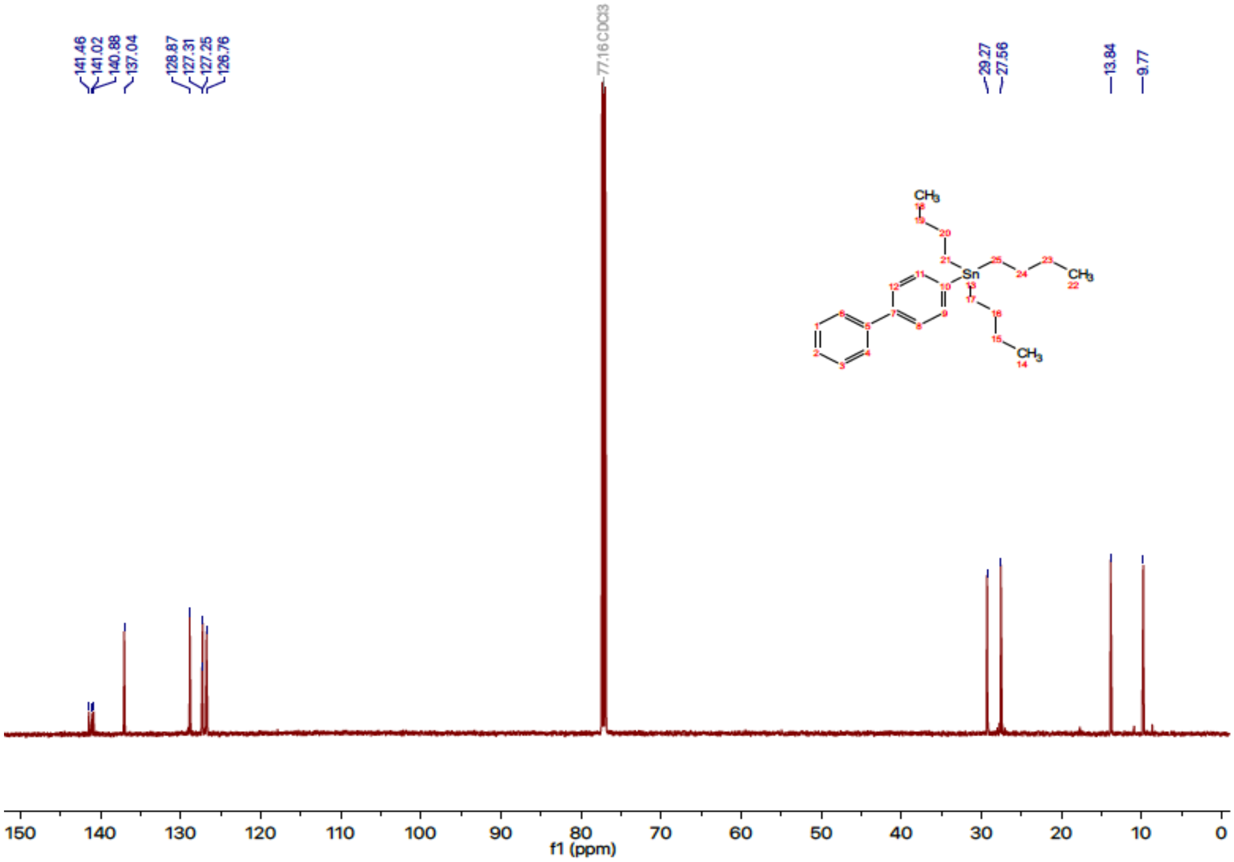

2.2. 2-((4-(tributylstannyl)phenyl)methoxy)pyrimidin-4-amine (3)

2.2.1. 1H NMR:

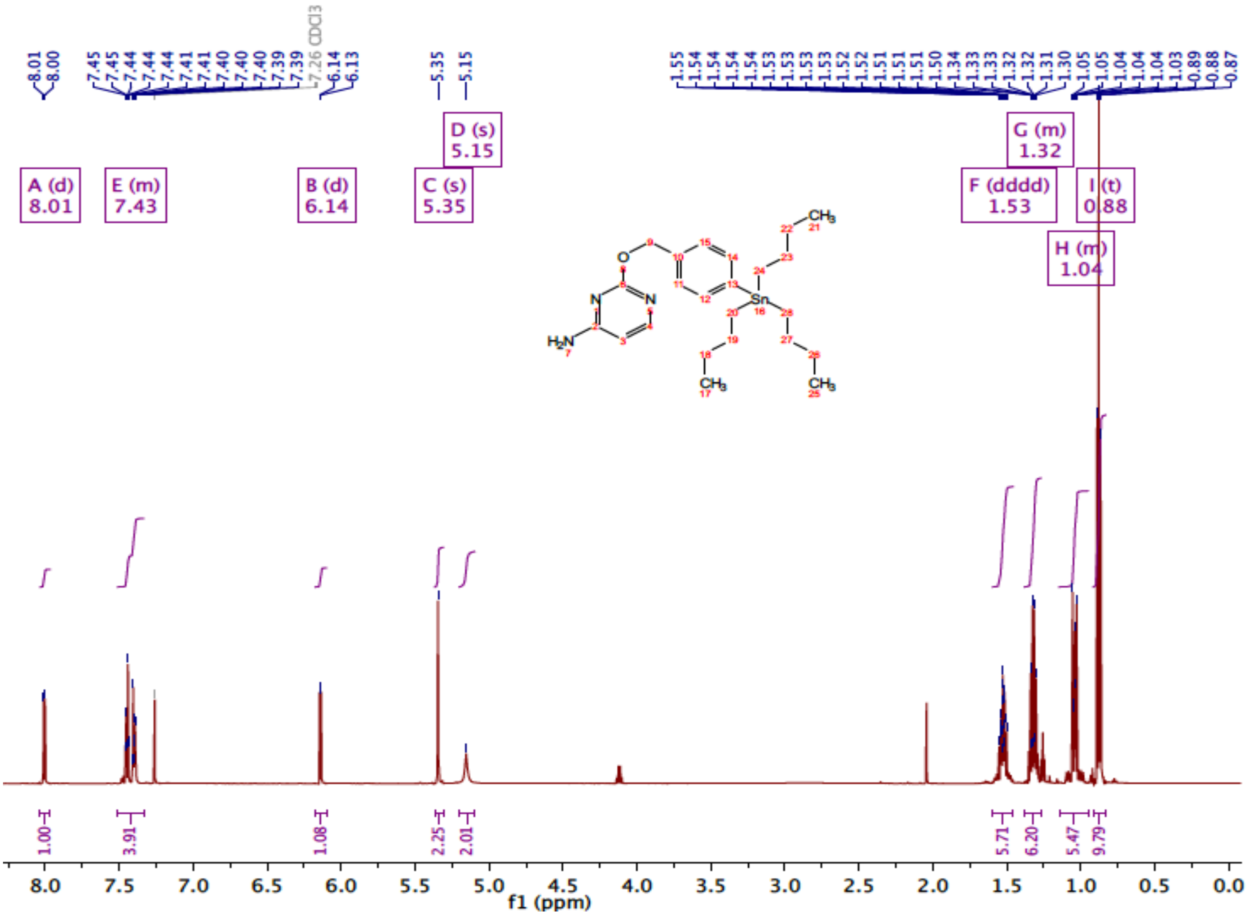

2.2.2. 13C NMR:

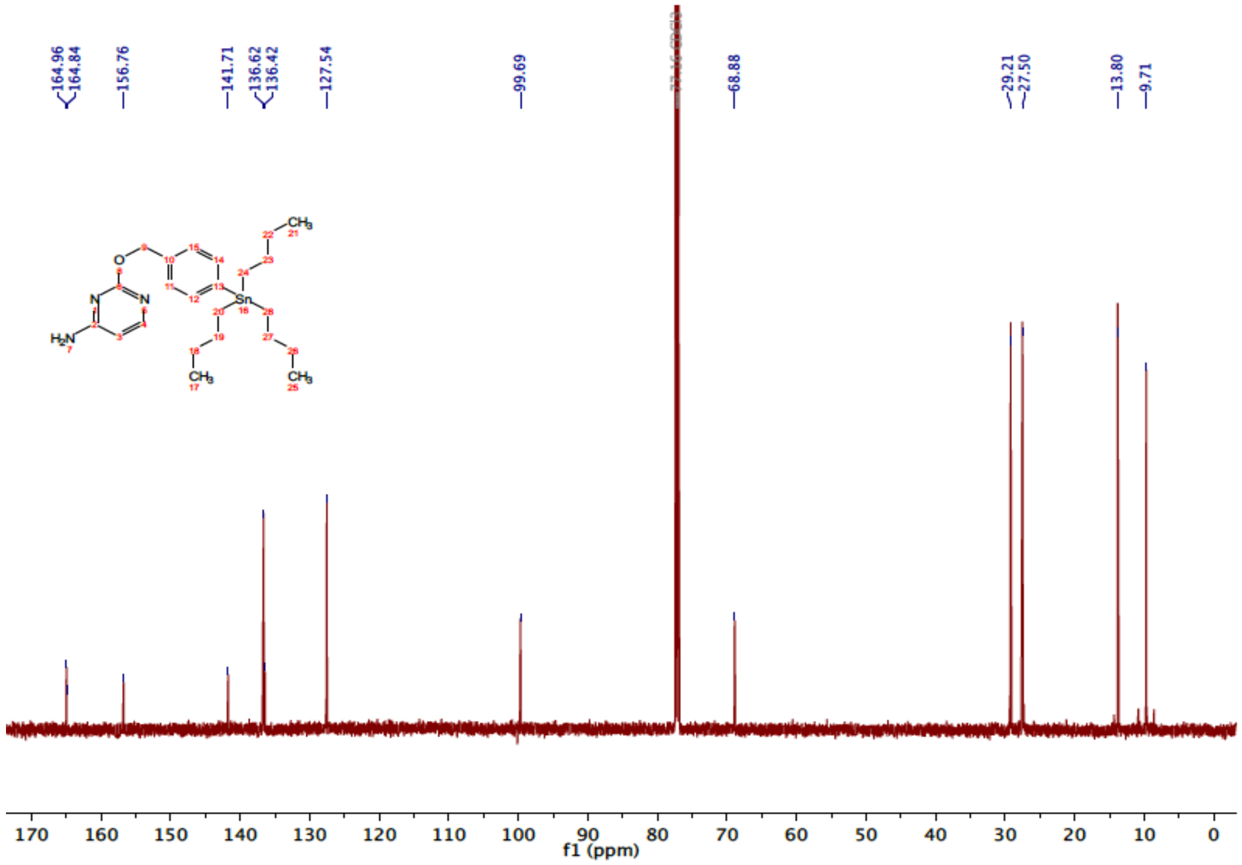

2.3. 2-((4-fluorophenyl)methoxy)pyrimidin-4-amine (4)

2.3.1. 1H NMR:

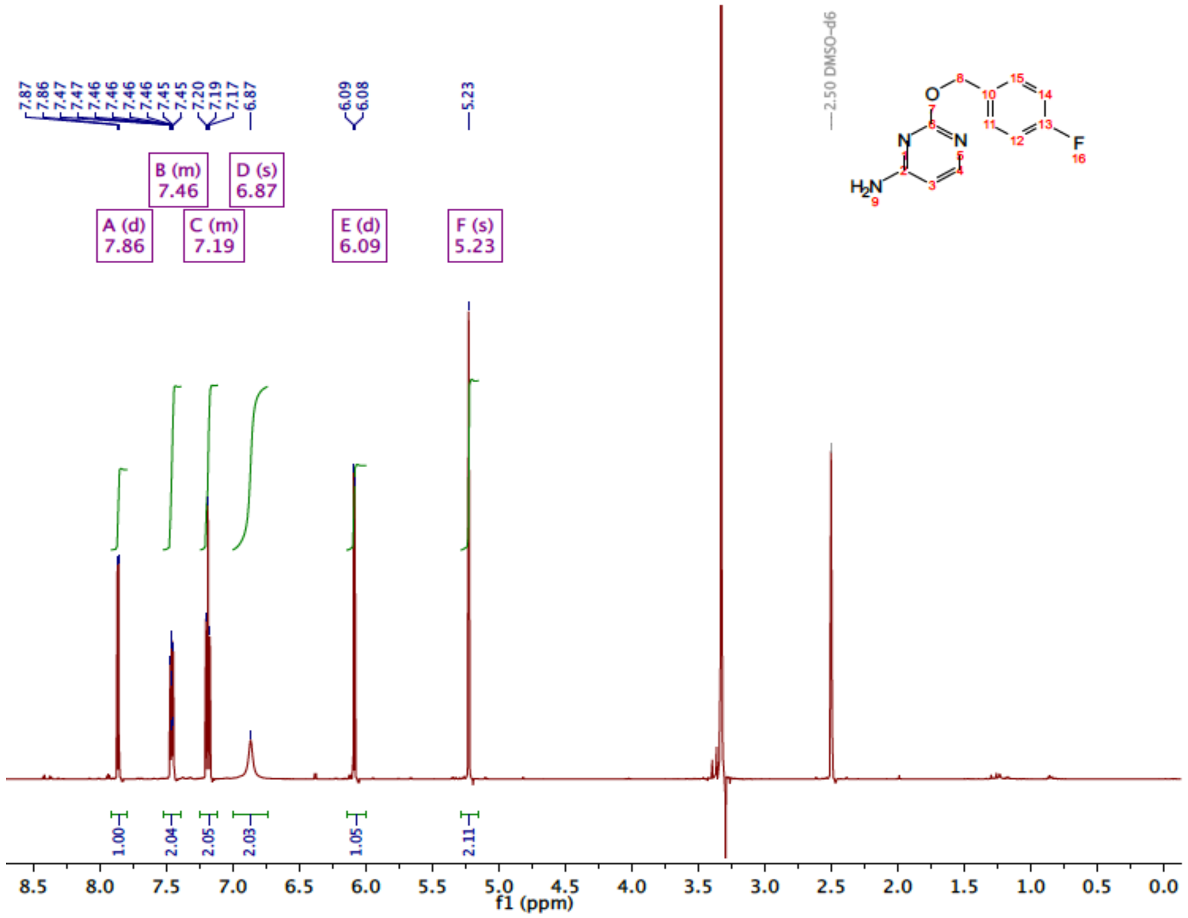

2.3.2. 13C NMR:

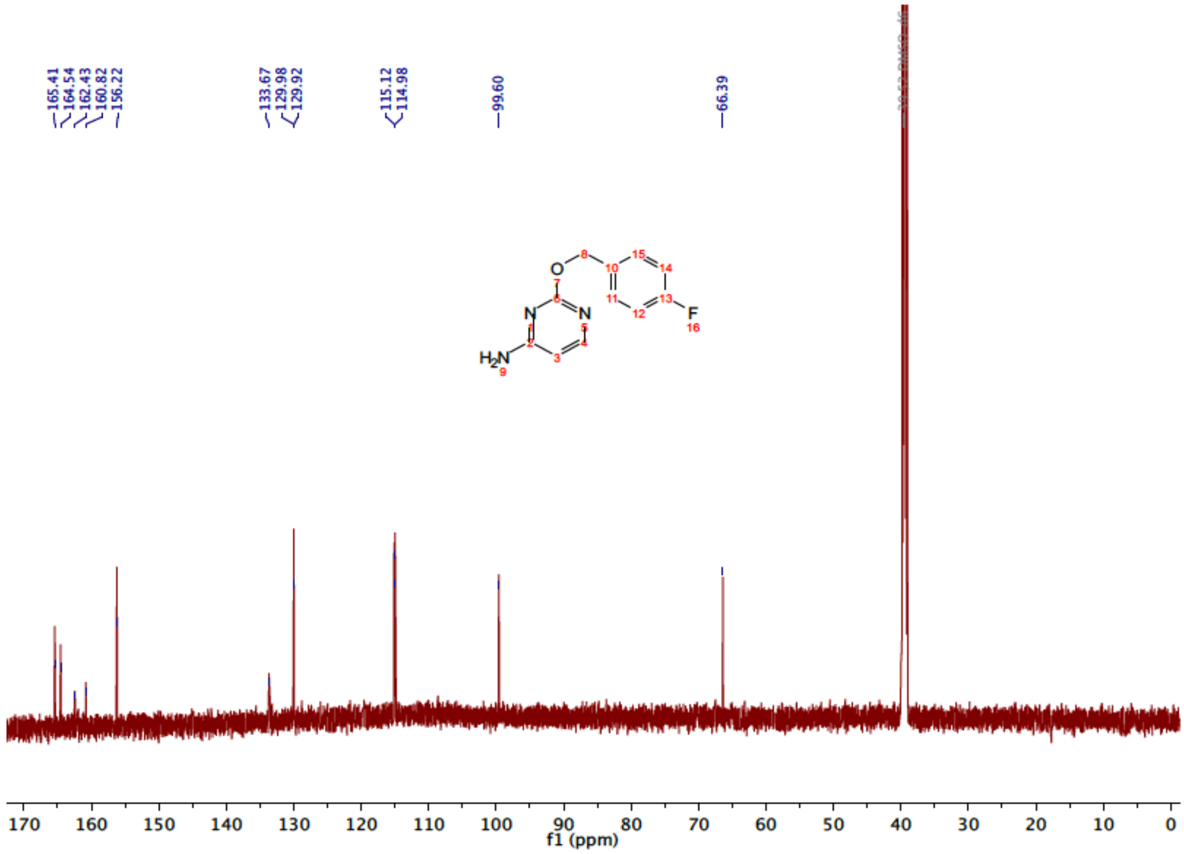

2.4. 4-(tributylstannyl)benzyl alcohol (5)

2.4.1. 1H NMR:

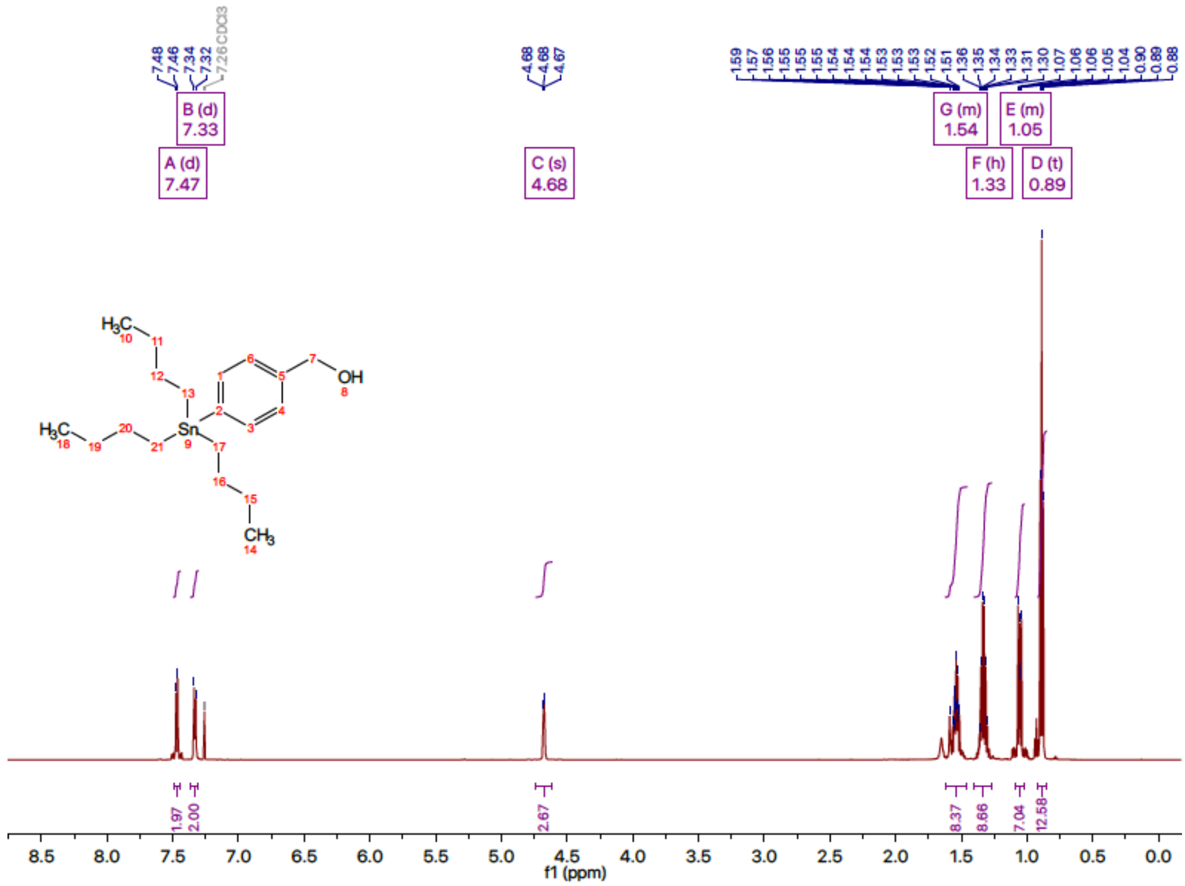

2.4.2. 13C NMR:

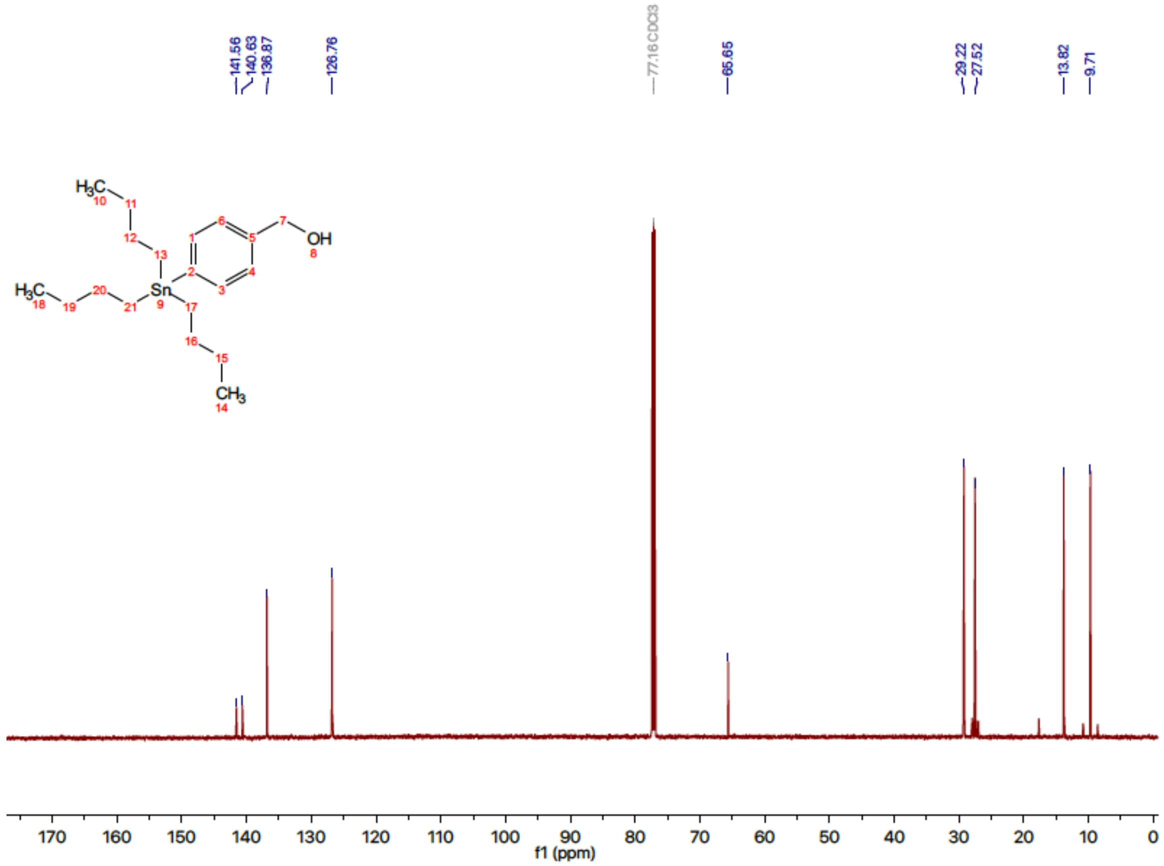

3. Radiochemistry:

3.1. General Procedure for DoE radiochemistry experiments

The general radiochemical procedure used throughout this study are described and discussed in the methods section of the manuscript.

3.2. Analytical HPLC Conditions:

All radioHPLCs were run with the following gradient:

Column: Luna 5µm C18 100Å. Column 250 x 4.6 mm.

Gradient of MeCN/H_2O + 0.1%TFA. Flow rate: 1 mL/min:

0-2 min: 5% MeCN
2-17 min: 5% to 100% MeCN
17-23 min: 100%
23-28 min: 100-5%

The following compounds were synthesized using the general procedure described in the manuscript:

3.3. Representative Radiochemical Analysis: 4- [^{18}F]fluorobiphenyl

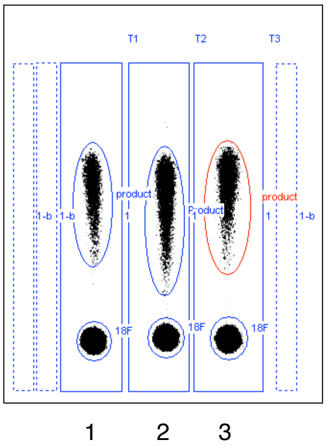

1

ID	Net % Max Reg.	Label
1 - Rect	100,0	
2 - Ell	43,0	product
3 - Ell	45,3	18F

2

ID	Net % Max Reg.	Label
1 - Rect	100,0	
2 - Ell	51,1	Product
3 - Ell	39,1	18F

3

ID	Net % Max Reg.	Label
1 - Rect	100,0	
2 - Ell	47,8	product
3 - Ell	41,3	18F

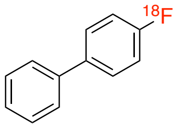

RadioTLC conditions:
Hexane/Ethyl Acetate 1:1

Sup.Figure 1: Representative radioTLC data for 4-[^{18}F]fluorobiphenyl

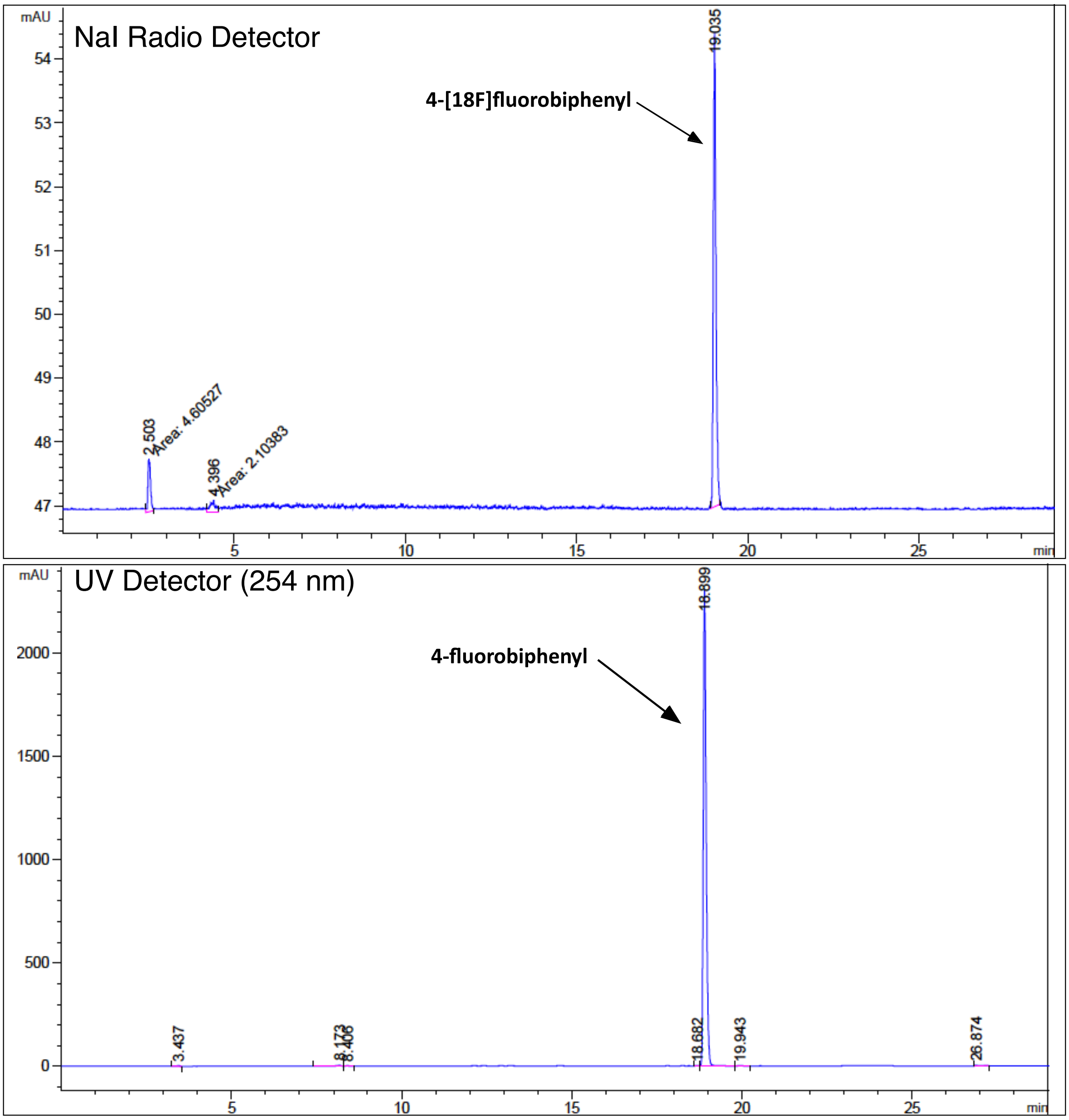

Sup.Figure 2: Representative radioHPLC data for 4-[^{18}F]fluorobiphenyl

3.4. Representative Radiochemical Analysis: 2-((4-[18F]fluorophenyl)methoxy)pyrimidin-4-amine ([18F]*p*FBC)

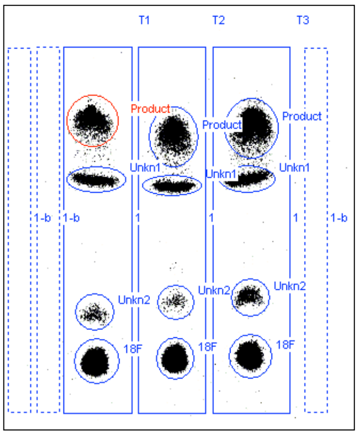

1

ID	Net % Max Reg.	Label
1 - Rect	100,0	
2 - Ell	21,5	Product
3 - Ell	51,4	18F
4 - Ell	11,5	Unkn1
5 - Ell	5,5	Unkn2

2

ID	Net % Max Reg.	Label
1 - Rect	100,0	
2 - Ell	24,6	Product
3 - Ell	50,3	18F
4 - Ell	12,3	Unkn1
5 - Ell	4,3	Unkn2

3

ID	Net % Max Reg.	Label
1 - Rect	100,0	
2 - Ell	28,9	Product
3 - Ell	44,9	18F
4 - Ell	11,2	Unkn1
5 - Ell	6,1	Unkn2

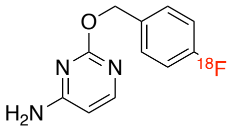

RadioTLC conditions:
Dichloromethane/
Metanol 1:1

Sup.Figure 3: Representative radioTLC data for [18F]pFBC.

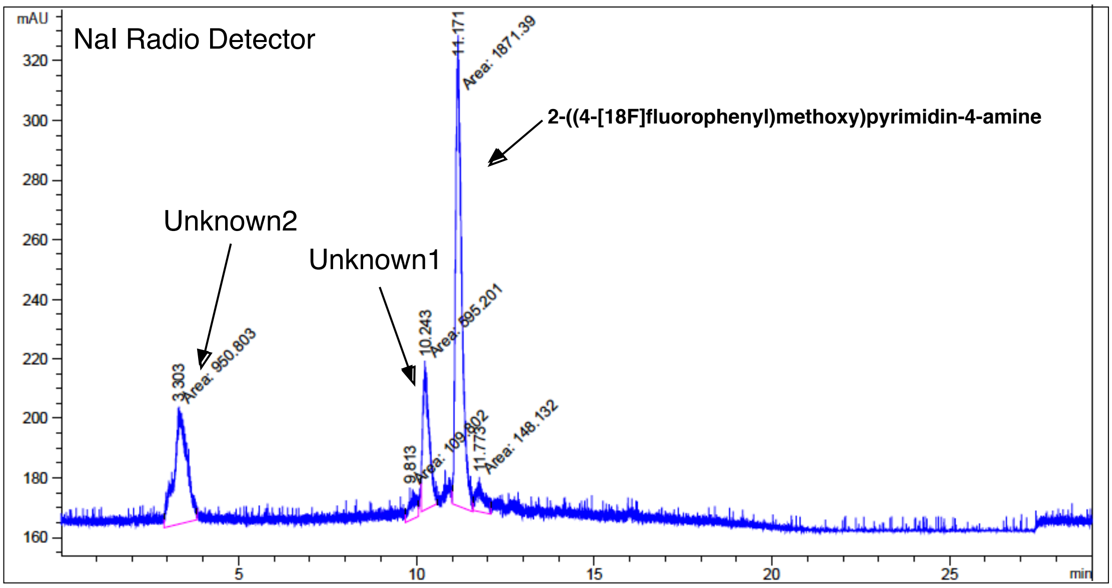

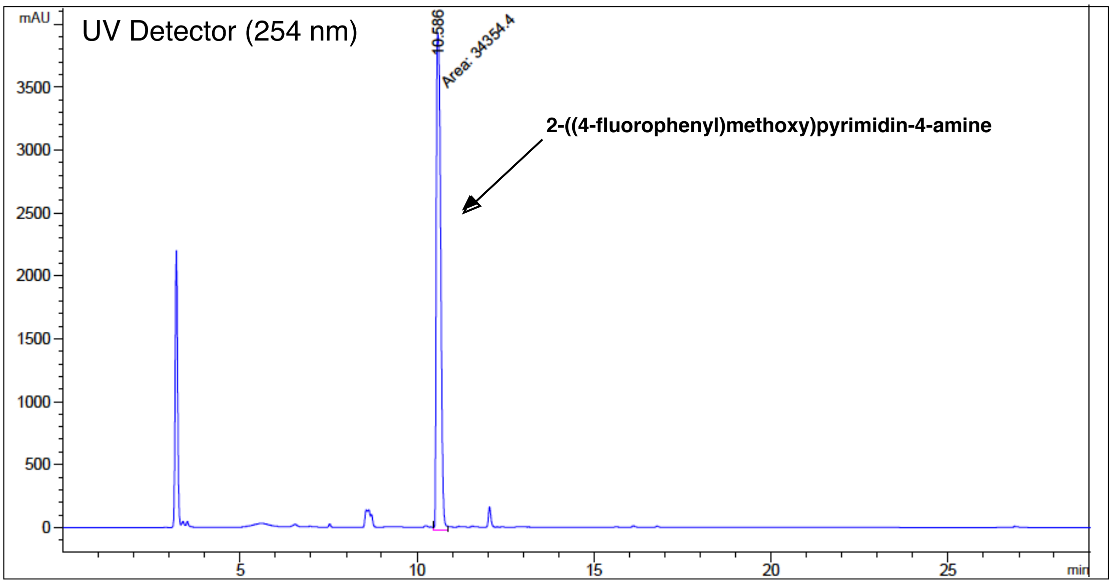

Sup.Figure 4: Representative radioHPLC data for [18F]pFBC.

3.5. Representative Radiochemical Analysis: 4-[^{18}F]fluorobenzyl alcohol ([^{18}F]*p*BnOH)

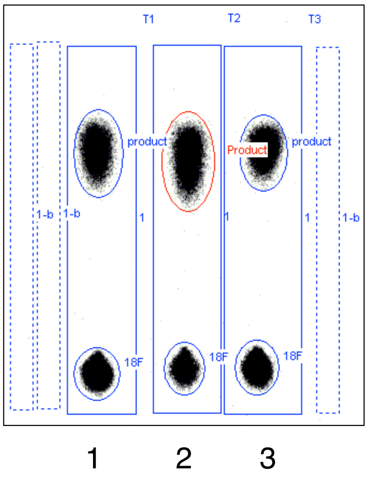

1

ID	Net % Max Reg.	Label
1 - Rect	100,0	
2 - Ell	53,3	product
3 - Ell	38,9	18F

2

ID	Net % Max Reg.	Label
1 - Rect	100,0	
2 - Ell	68,0	Product
3 - Ell	26,1	18F

3

ID	Net % Max Reg.	Label
1 - Rect	100,0	
2 - Ell	54,1	product
3 - Ell	38,2	18F

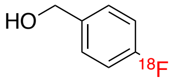

RadioTLC conditions:
Hexane/Ethyl Acetate 2:8

Sup.Figure 5: Representative radioTLC data for [^{18}F]pBnOH

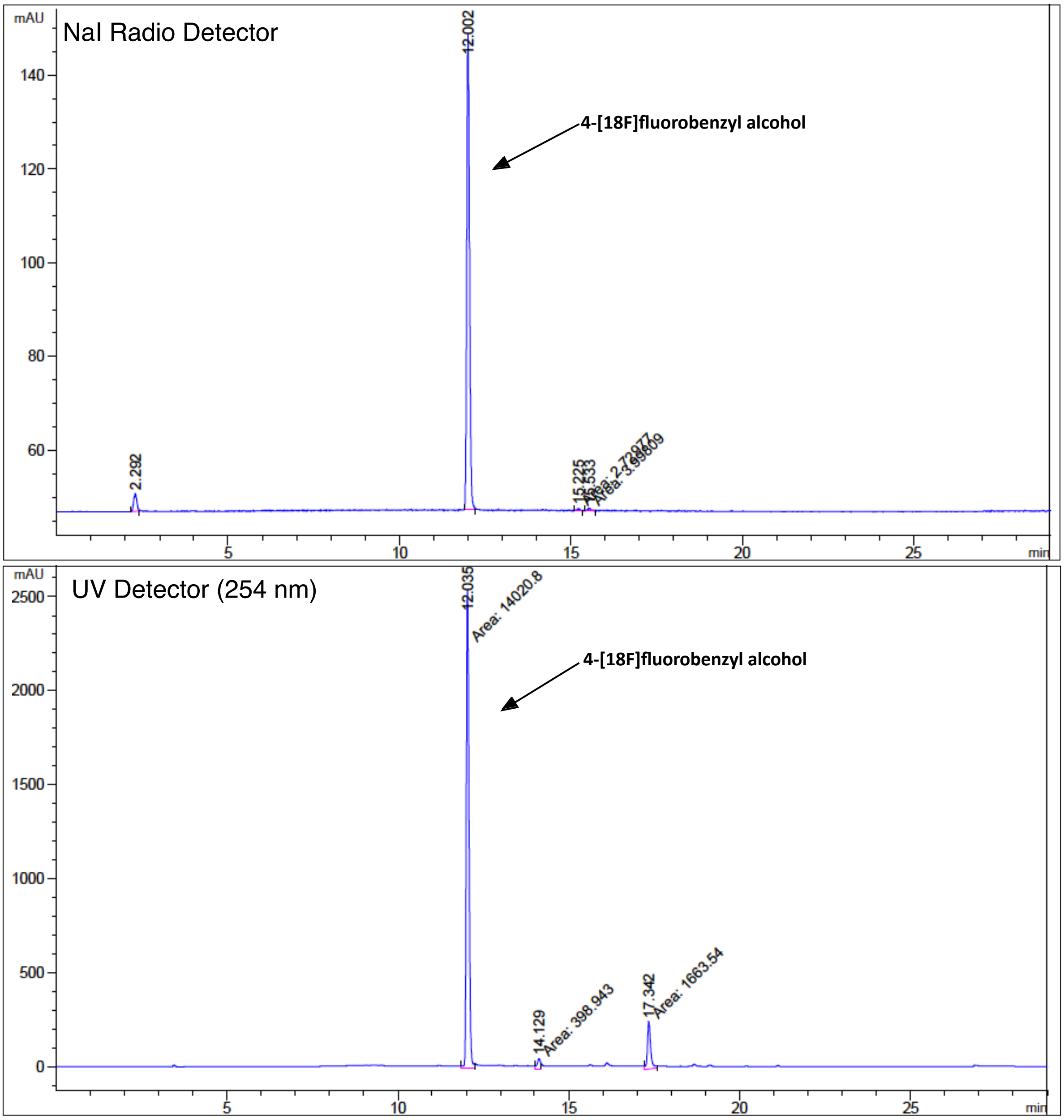

Sup.Figure 6: Representative radioHPLC data for [^{18}F]pBnOH.

4. DoE:

All DoE studies were designed using the DoE software package *Modde Go 12 (Umetrics)*. The "worksheet" table of experiments to be performed was used to calculate the amounts of the reagents needed for each individual run. All experiments were performed in randomized order but are listed here in an order representative of the structure of the experimental design (experimental points (blue), then starpoints (red), then centerpoints (green)). After the RCC (Y) data was collected it was transformed by 10Log (Y) to ensure the data set approximated a normal distribution.

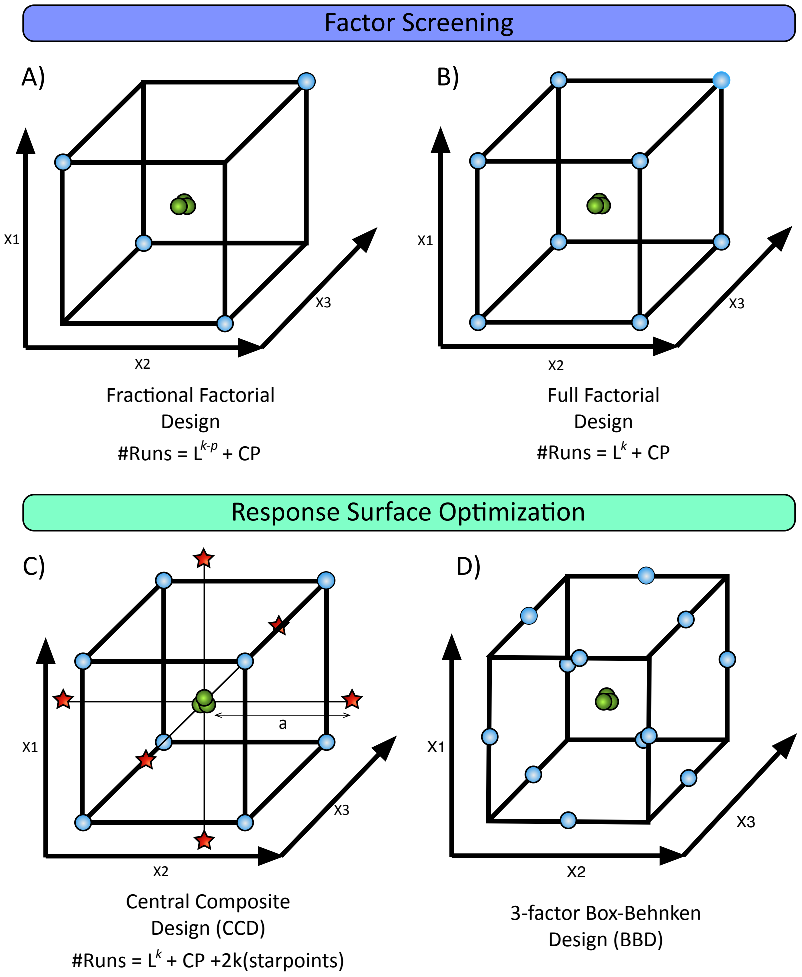

Sup. Figure 7: Common factorial experimental arrangements around a K-Dimensional hypercube (factorial points shown in blue) (K is the total number of factors, in this case K =3). L is the number of setting chosen (for most factorial designs, such as those pictured here L = 2.) For Fractional factorial designs (A), p is the total number of generators used to form the array ($1/K^p$ is the fraction of the total number of runs from the full factorial experiment (B)). Center points (CP, shown in Green) are repeated experiments carried out at the center of the hypercube to estimate reproducibility and measure curvature in the response surface. In a Central Composite Design (CCD), the starpoints (shown in red) are arranged around the faces of the cube (C). A Central Composite Face Centered design (CCF) has the starpoints positioned on the surface of the hypercube. A Central Composite Circumscribed design (CCC) has the starpoints positioned a distance "a" from the center of the hypercube so as to circumscribe a sphere around the factorial cube. An orthogonal central composite design (CCO) has a distance "a" so as to ensure orthogonality in the experimental matrix. An alternative to the CCD is the Box-Behnken Design (BBD), where experiment al points are on the edges of the process space cube (D). The (BBD) can be particularly useful when the process does not work well at the combined extremes of the experimental factors.

Sup.Table 1: The number of total experimental runs required for a selection of different experimental designs.

	Factor Screening Designs				Optimization	
	OVAT[a]	Fractional Factorial[c]			Full Factorial	Central Composite Design (CCD)[g]
		RES III[d]	RES IV[e]	RES V[f]		
Non-CP runs	NA	L^{k-p}	L^{k-p}	L^{k-p}	L^{k}	L^{k} + 2k(starpoints)
Settings per Factor (L)	3 - 6	2	2	2	2	5
Factors investigated (k)	8	9	8	8	8	3
Formula	NA	2_{III}^{9-5}	2_{IV}^{8-4}	2_{V}^{8-2}	2^{8}	$2^{3} + 6$
Repetitions/center-points	n=2[b]	Cp=3	Cp=3	Cp=3	Cp=3	Cp=3
Total number of runs	74	19	19	67	259	17

a) The results of this column were tallied from the paper by Makaravage et al.[11] b)The authors stated that n > 2 runs were performed for each experimental point. For this tally, it was assumed that each run was performed only twice. c) The number of runs for the corresponding fractional factorial RES III, RES IV, and RES V designs (with k factors and p generators) were taken from the table printed in Box, Hunter and Hunter 1979.[36] d) A resolution (RES) III fractional factorial design is defined as a design were all main effects are confounded with 2-factor interactions. e) A RES IV fractional factorial design is defined as a design were no main effects are confounded with 2-factor interactions, but all 2-factor interaction are confounded with each other. f) In a RES V fractional factorial design main effects and 2-factor interaction as not confounded but, 2-factor interactions are confounded with 3-factor interaction. g) A CCD is a response surface optimization design.

4.1. Factor Screening on the CMRF of a Model Arylstannane:

A fractional factorial experimental design consisting of 16 experimental points and 8 center points was constructed in 4 blocks (to model day-to-day experimental variances across 4 days). Each block consisted of 4 experimental runs and 2 center points. Each radiochemical experiment was conducted in accordance with the general procedure outlined above for 15 min. The obtained RCC data was transformed by 10Log(Y) to approximate a normal distribution of the data. The model was fitted using multiple linear regression (MLR).

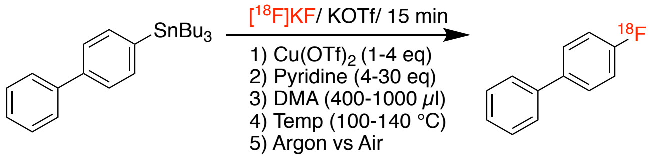

Sup. Table 2: DoE factor screening experimental table for the model radiofluorination of 4-(tributylstannyl)biphenyl. Experimental points are in blue, centerpoints are in green.

Exp No	Temp	DMA Volume (µl)	Catalyst Loading (eq)	Catalyst mmol	Catalyst mass (mg)	Pyridine Loading (eq)	Pyridine mmol	Pyridine mass (mg)	Pyridine Volume (ul)	Atmosphere	Block	RCC
1	100	400	1	0.0045	1.63	4	0.02	1.43	1.40	Air	B1	3
2	140	400	4	0.0180	6.50	4	0.02	1.43	1.40	Air	B1	10.9
3	140	1000	1	0.0045	1.63	30	0.14	10.69	10.50	Argon	B1	7
4	100	1000	4	0.0180	6.50	30	0.14	10.69	10.50	Argon	B1	31.2
5	120	700	2.5	0.0113	4.07	17	0.08	6.06	5.95	Argon	B1	22
6	120	700	2.5	0.0113	4.07	17	0.08	6.06	5.95	Argon	B1	33
7	100	1000	1	0.0045	1.63	4	0.02	1.43	1.40	Argon	B2	5.7
8	140	1000	4	0.0180	6.50	4	0.02	1.43	1.40	Argon	B2	6
9	140	400	1	0.0045	1.63	30	0.14	10.69	10.50	Air	B2	10.2
10	100	400	4	0.0180	6.50	30	0.14	10.69	10.50	Air	B2	23.2
11	120	700	2.5	0.0113	4.07	17	0.08	6.06	5.95	Argon	B2	35
12	120	700	2.5	0.0113	4.07	17	0.08	6.06	5.95	Argon	B2	23
13	140	1000	1	0.0045	1.63	4	0.02	1.43	1.40	Air	B3	5.9
14	100	1000	4	0.0180	6.50	4	0.02	1.43	1.40	Air	B3	13
15	100	400	1	0.0045	1.63	30	0.14	10.69	10.50	Argon	B3	8.3
16	140	400	4	0.0180	6.50	30	0.14	10.69	10.50	Argon	B3	33
17	120	700	2.5	0.0113	4.07	17	0.08	6.06	5.95	Argon	B3	31.2
18	120	700	2.5	0.0113	4.07	17	0.08	6.06	5.95	Argon	B3	36.7
19	140	400	1	0.0045	1.63	4	0.02	1.43	1.40	Argon	B4	2.1
20	100	400	4	0.0180	6.50	4	0.02	1.43	1.40	Argon	B4	19.6
21	100	1000	1	0.0045	1.63	30	0.14	10.69	10.50	Air	B4	12.3
22	140	1000	4	0.0180	6.50	30	0.14	10.69	10.50	Air	B4	15.9
23	120	700	2.5	0.0113	4.07	17	0.08	6.06	5.95	Argon	B4	29
24	120	700	2.5	0.0113	4.07	17	0.08	6.06	5.95	Argon	B4	29

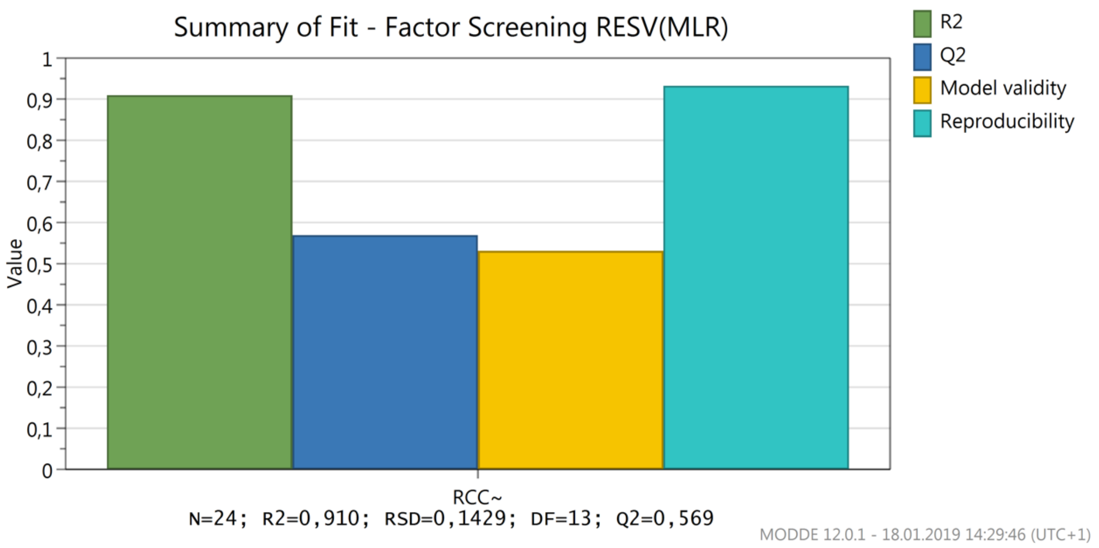

Sup.Figure 8: Summary statistics for the factor screening DoE of the model radiofluorination of 4-(tributylstannyl)biphenyl. R^2 represents the goodness of regression model fit. Q^2 represents the goodness of model prediction. "Reproducibility" is calculated from the standard deviation in replicate (centerpoint) experiment results.

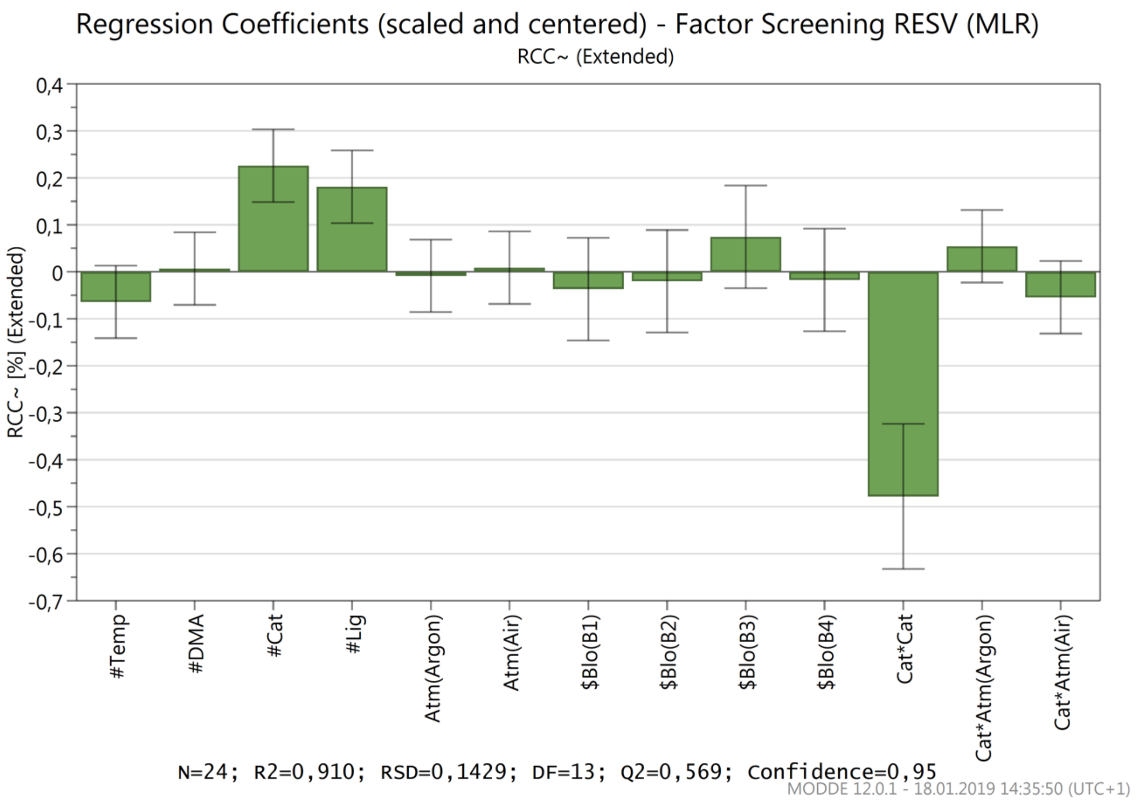

Sup.Figure 9: The scaled and centered regression factors calculated from the results of fractional factorial factor screening DoE. Large bars represent factors with a large contribution to the response (%RCC). A positive number denoted as positive influence on the response. A negative number denotes a diminishing effect on the response. If a factor's regression coefficient is smaller than the associated errors bars it is probable (at the 95% confidence interval) that that factor is not significant.

4.2. Response Surface Optimization For the synthesis of [^{18}F]pFBC

A orthogonal central composite (CCO) experimental design consisting of 14 experimental points and 3 center points was constructed (17 runs total). The investigated factors were precursor loading (10 - 30 µmol),

$Cu(OTf)_2$ (1 -4 eq), and pyridine loading (10 - 40 eq). All reactions were performed in 700 µl of DMA at 110°C for 15 min according to the general procedure outlined above. The RCC data was obtained from radioTLC and was transformed by 10Log(Y) to approximate a normal distribution of the data. The model was then fitted using multiple linear regression (MLR)

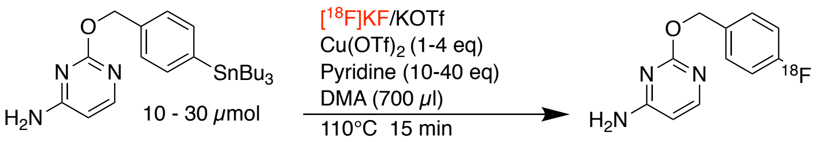

Sup. Table 3: DoE response surface optimization (CCO) experimental table for the radiosynthesis [18F]pFBC. Experimental points are in blue, Starpoints are in red, centerpoints are in green.

Exp No	Catalyst Load (mg)	Catalyst mmol	Catalyst Mass (mg)	Pyridine Load (eq)	Pyridine mmol	Pyridine Mass (mg)	Pyridine Vol (µl)	Substrate Load (µmol)	Substrate Mass (mg)	DMA total	DMA Vol (ul)	RCC
1	1	0.010	3.6	10	0.10	7.9	8.1	10	4.9	700	607	**2.1**
2	4	0.040	14.4	10	0.10	7.9	8.1	10	4.9	700	499	**18.5**
3	1	0.010	3.6	40	0.40	31.6	32.2	10	4.9	700	583	**4.2**
4	4	0.040	14.4	40	0.40	31.6	32.2	10	4.9	700	474	**14.2**
5	1	0.030	10.8	10	0.30	23.7	24.2	30	14.7	700	421	**6.6**
6	4	0.120	43.3	10	0.30	23.7	24.2	30	14.7	700	96	**17.8**
7	1	0.030	10.8	40	1.20	94.9	96.7	30	14.7	700	348	**0.4**
8	4	0.120	43.3	40	1.20	94.9	96.7	30	14.7	700	23	**1.1**
9	0.47031	0.009	3.4	25	0.50	39.6	40.3	20	9.8	700	528	**2.7**
10	4.5297	0.091	32.7	25	0.50	39.6	40.3	20	9.8	700	235	**17.3**
11	2.5	0.050	18.1	4.70305	0.09	7.4	7.6	20	9.8	700	414	**9.4**
12	2.5	0.050	18.1	45.297	0.91	71.7	73.0	20	9.8	700	349	**1.5**
13	2.5	0.016	5.8	25	0.16	12.8	13.0	6.46890	3.2	700	597	**30.5**
14	2.5	0.084	30.3	25	0.84	66.3	67.5	33.5313	16.4	700	166	**5.9**
15	2.5	0.050	18.1	25	0.50	39.6	40.3	20	9.8	700	381	**11.4**
16	2.5	0.050	18.1	25	0.50	39.6	40.3	20	9.8	700	381	**15.0**
17	2.5	0.050	18.1	25	0.50	39.6	40.3	20	9.8	700	381	**21.3**

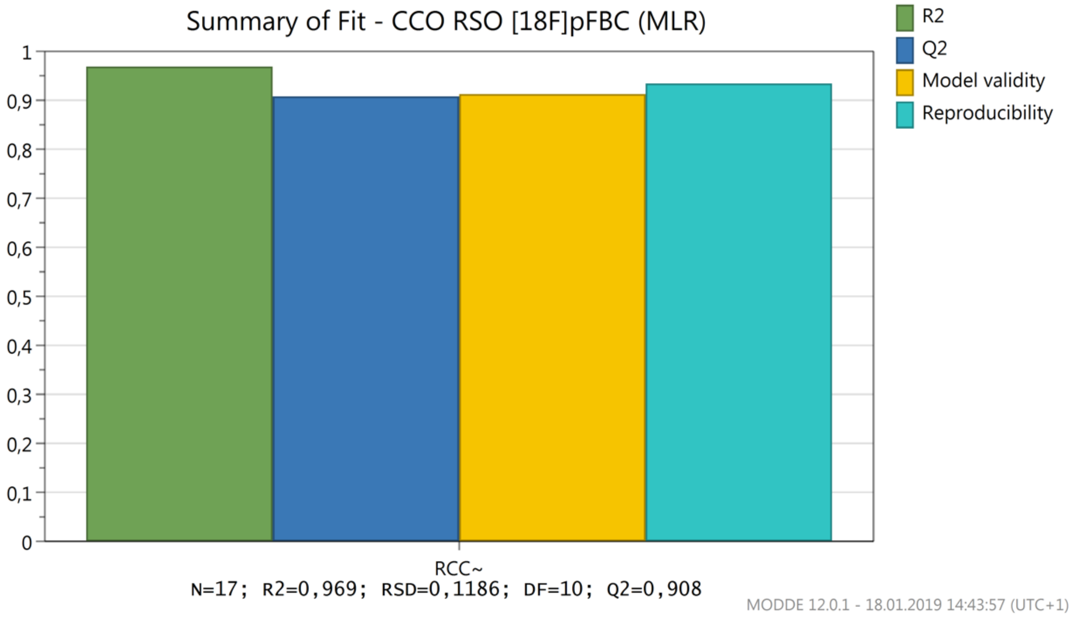

Sup.Figure 10: Summary statistics for DoE response surface optimization (BBD) experimental table for the radiofluorination [18F]pFBC. R^2 represents the goodness of regression model fit. Q^2 represents the goodness of model prediction. “Reproducibility” is calculated from the standard deviation in replicate (centerpoint) experiment results.

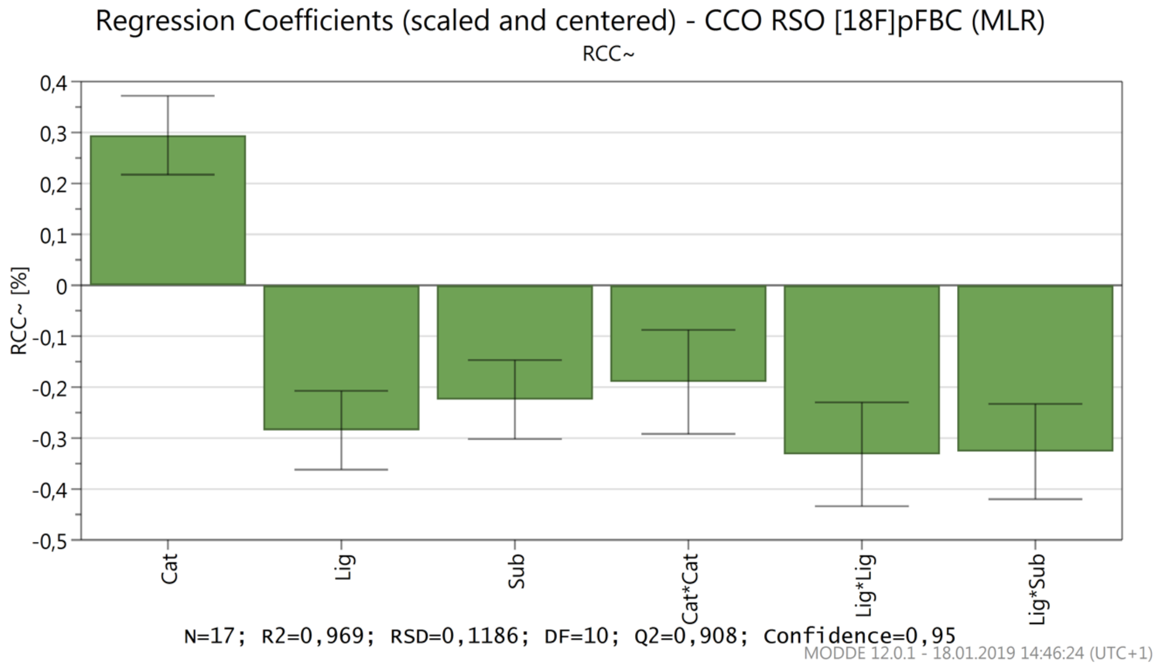

Sup.Figure 11: The scaled and centered regression factors calculated from the DoE response surface optimization (CCO) experimental table for the radiosynthesis of [18F]pFBC. Large bars represent factors with a large contribution to the response (%RCC). A positive number denoted as positive influence on the response. A negative number denotes a diminishing effect on the response. If a factor’s regression coefficient is smaller than the associated errors bars it is probable (at the 95% confidence interval) that that factor is not significant.

4.3. Response Surface Optimization or the Synthesis of [18F]FBnOH:

A Box-Behnken design (BBD) composed of 12 experimental points and 3 center points was constructed (15 runs total). The investigated factors were precursor loading (5 - 25 µmol), $Cu(OTf)_2$ (1 - 4 eq), and pyridine loading (5 - 30 eq). All reactions were performed in 700 µl of DMA at 110°C for 20 min according to the

general procedure outlined above. The RCC data was obtained via radioTLC and used directly for regression modelling without further manipulation.

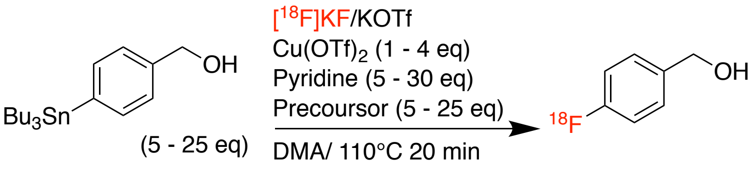

Sup. Table 4: DoE response surface optimization (BBD) experimental table for the radiofluorination of 4-(tributylstannyl)benzyl alcohol. Experimental points are in blue, centerpoints are in green.

Exp No	Catalyst Load (eq)	Catalyst (mmol)	Catalyst Mass (mg)	Pyridine Load (eq)	Pyridine (mmol)	Pyridine Mass (mg)	Pyridine Vol (µl)	Substrate Load (µmol)	Substrate Mass (mg)	DMA total	DMA Vol (ul)	RCC
1	1	0.015	5.4	5	0.08	5.9	6.0	15	6.0	700	580	**49.6**
2	4	0.060	21.7	5	0.08	5.9	6.0	15	6.0	700	418	**65.3**
3	1	0.015	5.4	30	0.45	35.6	36.2	15	6.0	700	550	**18.3**
4	4	0.060	21.7	30	0.45	35.6	36.2	15	6.0	700	388	**44.1**
5	1	0.005	1.8	17.5	0.09	6.9	7.0	5	2.0	700	655	**26.9**
6	4	0.020	7.2	17.5	0.09	6.9	7.0	5	2.0	700	601	**65.1**
7	1	0.025	9.0	17.5	0.44	34.6	35.2	25	9.9	700	475	**29.5**
8	4	0.100	36.1	17.5	0.44	34.6	35.2	25	9.9	700	205	**56.6**
9	2.5	0.013	4.5	5	0.03	2.0	2.0	5	2.0	700	633	**43.5**
10	2.5	0.013	4.5	30	0.15	11.9	12.1	5	2.0	700	623	**39**
11	2.5	0.063	22.6	5	0.13	9.9	10.1	25	9.9	700	365	**65.1**
12	2.5	0.063	22.6	30	0.75	59.3	60.4	25	9.9	700	315	**14.7**
13	2.5	0.038	13.5	17.5	0.26	20.8	21.1	15	6.0	700	484	**52.4**
14	2.5	0.038	13.5	17.5	0.26	20.8	21.1	15	6.0	700	484	**54.2**
15	2.5	0.038	13.5	17.5	0.26	20.8	21.1	15	6.0	700	484	**52.3**

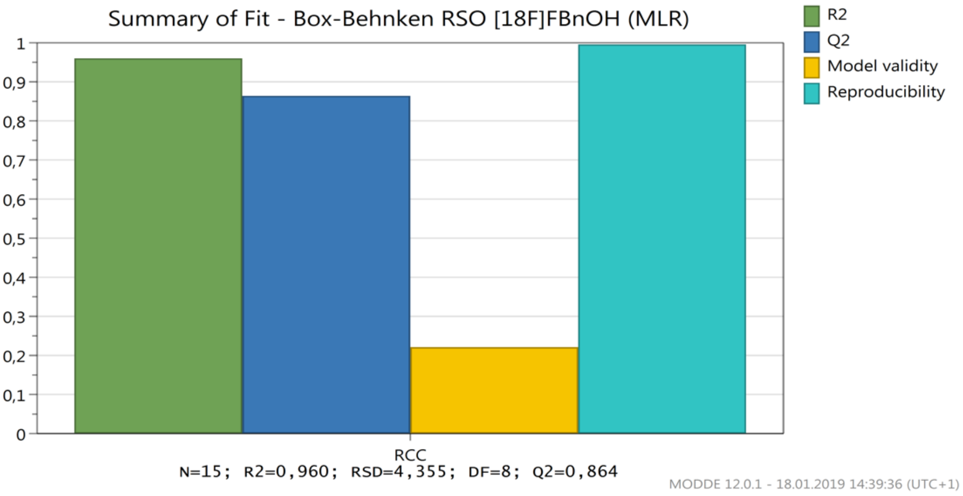

Sup.Figure 12: Summary statistics for the DoE response surface optimization (BBD) experimental table for the radiofluorination of 4-(tributylstannyl)benzyl alcohol. R^2 represents the goodness of regression model fit. Q^2 represents the goodness of model prediction. “Reproducibility” is calculated from the standard deviation in replicate (centerpoint) experiment results. The low “model validity” in this case is due to the high reproducibility term; as R^2 and Q^2 are both acceptably high and in agreement the model can still be considered to be valid and useful.

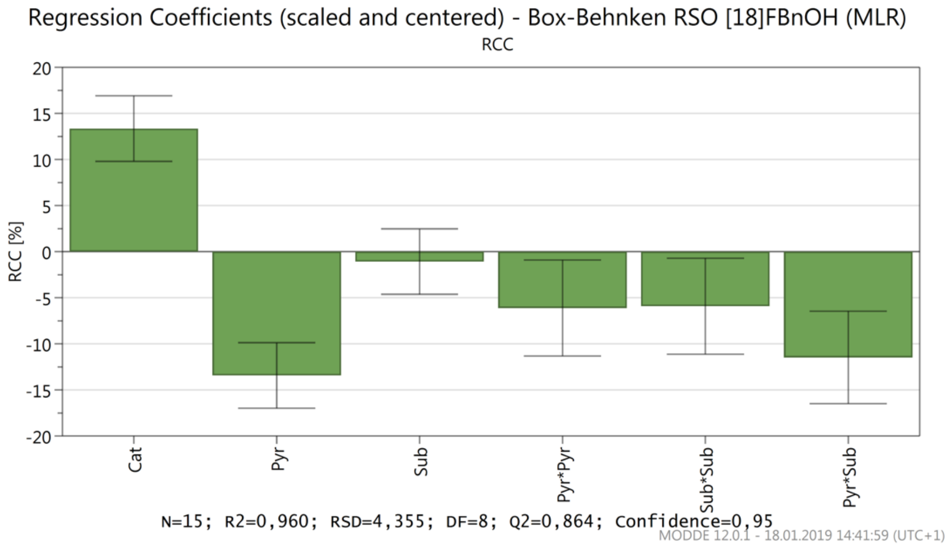

Sup.Figure 13: The scaled and centered regression factors calculated from the DoE response surface optimization (BBD) experimental table for the radiofluorination of 4-(tributylstannyl)benzyl alcohol. Large bars represent factors with a large contribution to the response (%RCC). A positive number denoted as positive influence on the response. A negative number denotes a diminishing effect on the response. If a factor's regression coefficient is smaller than the associated errors bars it is probable (at the 95% confidence interval) that that factor is not significant.

5. References

1. Makaravage, K. J., Brooks, A. F., Mossine, A. V., Sanford, M. S. & Scott, P. J. H. H. Copper-Mediated Radiofluorination of Arylstannanes with [18F]KF. *Org. Lett.* **18**, 5440–5443 (2016).

2. Kopka, K. *et al.* 5-Pyrrolidinylsulfonyl isatins as a potential tool for the molecular imaging of caspases in apoptosis. *J. Med. Chem.* **49**, 6704–6715 (2006).
